# Candidate locus analysis of the *TERT*–*CLPTM1L* cancer risk region on chromosome 5p15 identifies multiple independent variants associated with endometrial cancer risk

**DOI:** 10.1007/s00439-014-1515-4

**Published:** 2014-12-09

**Authors:** Luis G. Carvajal-Carmona, Tracy A. O’Mara, Jodie N. Painter, Felicity A. Lose, Joe Dennis, Kyriaki Michailidou, Jonathan P. Tyrer, Shahana Ahmed, Kaltin Ferguson, Catherine S. Healey, Karen Pooley, Jonathan Beesley, Timothy Cheng, Angela Jones, Kimberley Howarth, Lynn Martin, Maggie Gorman, Shirley Hodgson, National Study of Endometrial Cancer Genetics Group (NSECG), The Australian National Endometrial Cancer Study Group (ANECS), Nicholas Wentzensen, Peter A. Fasching, Alexander Hein, Matthias W. Beckmann, Stefan P. Renner, Thilo Dörk, Peter Hillemanns, Matthias Dürst, Ingo Runnebaum, Diether Lambrechts, Lieve Coenegrachts, Stefanie Schrauwen, Frederic Amant, Boris Winterhoff, Sean C. Dowdy, Ellen L. Goode, Attila Teoman, Helga B. Salvesen, Jone Trovik, Tormund S. Njolstad, Henrica M. J. Werner, Rodney J. Scott, Katie Ashton, Tony Proietto, Geoffrey Otton, Ofra Wersäll, Miriam Mints, Emma Tham, RENDOCAS, Per Hall, Kamila Czene, Jianjun Liu, Jingmei Li, John L. Hopper, Melissa C. Southey, Australian Ovarian Cancer Study (AOCS), Arif B. Ekici, Matthias Ruebner, Nichola Johnson, Julian Peto, Barbara Burwinkel, Frederik Marme, Hermann Brenner, Aida K. Dieffenbach, Alfons Meindl, Hiltrud Brauch, The GENICA Network, Annika Lindblom, Jeroen Depreeuw, Matthieu Moisse, Jenny Chang-Claude, Anja Rudolph, Fergus J. Couch, Janet E. Olson, Graham G. Giles, Fiona Bruinsma, Julie M. Cunningham, Brooke L. Fridley, Anne-Lise Børresen-Dale, Vessela N. Kristensen, Angela Cox, Anthony J. Swerdlow, Nicholas Orr, Manjeet K. Bolla, Qin Wang, Rachel Palmieri Weber, Zhihua Chen, Mitul Shah, Paul D. P. Pharoah, Alison M. Dunning, Ian Tomlinson, Douglas F. Easton, Amanda B. Spurdle, Deborah J. Thompson

**Affiliations:** 1 Genome Center and Department of Biochemistry and Molecular Medicine, School of Medicine, University of California, Davis, CA 95616 USA; 2 QIMR Berghofer Medical Research Institute, Brisbane, QLD Australia; 3 Department of Public Health and Primary Care, Centre for Cancer Genetic Epidemiology, University of Cambridge, Cambridge, UK; 4 Department of Oncology, Centre for Cancer Genetic Epidemiology, University of Cambridge, Cambridge, UK; 5 Wellcome Trust Centre for Human Genetics, University of Oxford, Oxford, UK; 6 Department of Clinical Genetics, St George’s Hospital Medical School, London, UK; 7 Division of Cancer Epidemiology and Genetics, National Cancer Institute, Bethesda, USA; 8 Division of Hematology/Oncology, Department of Medicine, David Geffen School of Medicine, University of California at Los Angeles, Los Angeles, CA UK; 9 Department of Gynecology and Obstetrics, Friedrich-Alexander University Erlangen-Nuremberg, University Hospital Erlangen, Erlangen, Germany; 10 Gynaecology Research Unit, Hannover Medical School, Hannover, Germany; 11 Clinics of Gynaecology and Obstetrics, Hannover Medical School, Hannover, Germany; 12 Department of Gynaecology, Jena University Hospital-Friedrich Schiller University, Jena, Germany; 13 Vesalius Research Center, VIB, Leuven, Belgium; 14 Department of Oncology, Laboratory for Translational Genetics, KU Leuven, Leuven, Belgium; 15 Department of Oncology, KU Leuven, Leuven, Belgium; 16 Division of Gynaecological Oncology, University Hospital Leuven, Leuven, Belgium; 17 Division of Gynecologic Oncology, Department of Obstetrics and Gynecology, Mayo Clinic, Rochester, MN USA; 18 Division of Epidemiology, Department of Health Science Research, Mayo Clinic, Rochester, MN USA; 19 Department of Clinical Science, Centre for Cancerbiomarkers, The University of Bergen, Bergen, Norway; 20 Department of Obstetrics and Gynecology, Haukeland University Hospital, Bergen, Norway; 21 Hunter Medical Research Institute, John Hunter Hospital, Newcastle, NSW Australia; 22 Hunter Area Pathology Service, John Hunter Hospital, Newcastle, NSW Australia; 23 Centre for Information Based Medicine, School of Biomedical Science and Pharmacy, University of Newcastle, Newcastle, NSW Australia; 24 Discipline of Medical Genetics, School of Biomedical Sciences and Pharmacy, University of Newcastle, Newcastle, NSW Australia; 25 School of Medicine and Public Health, University of Newcastle, Newcastle, NSW Australia; 26 Department of Women’s and Children’s Health, Karolinska Institutet, Karolinska University Hospital, Stockholm, Sweden; 27 Department of Molecular Medicine and Surgery, Karolinska Institutet, Stockholm, Sweden; 28 Department of Medical Epidemiology and Biostatistics, Karolinska Institutet, Stockholm, Sweden; 29 Human Genetics, Genome Institute of Singapore, Singapore, Singapore; 30 Centre for Epidemiology and Biostatistics, Melbourne School of Population and Global Health, The University of Melbourne, Melbourne, VIC Australia; 31 Department of Pathology, Genetic Epidemiology Laboratory, The University of Melbourne, Melbourne, VIC Australia; 32 Peter MacCullum Cancer Centre, Melbourne, VIC Australia; 33 Breakthrough Breast Cancer Research Centre, Institute of Cancer Research, London, UK; 34 London School of Hygiene and Tropical Medicine, London, UK; 35 Molecular Biology of Breast Cancer, Department of Gynecology and Obstetrics, University of Heidelberg, Heidelberg, Germany; 36 Division of Clinical Epidemiology and Aging Research, German Cancer Research Center (DKFZ), Heidelberg, Germany; 37 German Cancer Consortium (DKTK), Heidelberg, Germany; 38 Division of Tumor Genetics, Department of Obstetrics and Gynecology, Technical University of Munich, Munich, Germany; 39 Dr. Margarete Fischer-Bosch Institute of Clinical Pharmacology Stuttgart, University of Tuebingen, Tuebingen, Germany; 40 Institute for Occupational Medicine and Maritime Medicine, University Medical Center Hamburg-Eppendorf, Hamburg, Germany; 41 Department of Internal Medicine, Evangelische Kliniken Bonn gGmbH, Johanniter Krankenhaus, Bonn, Germany; 42 Institute of Pathology, Medical Faculty of the University of Bonn, Bonn, Germany; 43 Institute for Prevention and Occupational Medicine of the German Social Accident Insurance, Institute of the Ruhr University Bochum (IPA), Bochum, Germany; 44 Molecular Genetics of Breast Cancer, Deutsches Krebsforschungszentrum (DKFZ), Heidelberg, Germany; 45 Division of Cancer Epidemiology, German Cancer Research Center, Heidelberg, Germany; 46 Department of Cancer Epidemiology/Clinical Cancer Registry and Institute for Medical Biometrics and Epidemiology, University Clinic Hamburg-Eppendorf, Hamburg, Germany; 47 Department of Laboratory Medicine and Pathology, Mayo Clinic, Rochester, MN USA; 48 Department of Health Science Research, Mayo Clinic, Rochester, MN USA; 49 Cancer Epidemiology Centre, Cancer Council Victoria, Melbourne, Australia; 50 Department of Epidemiology and Preventive Medicine, Monash University, Melbourne, Australia; 51 Department of Biostatistics, University of Kansas Medical Center, Kansas City, KS USA; 52 Department of Genetics, Institute for Cancer Research, The Norwegian Radium Hospital, Oslo, Norway; 53 Faculty of Medicine, The K.G. Jebsen Center for Breast Cancer Research, Institute for Clinical Medicine, University of Oslo, Oslo, Norway; 54 Division of Medicine, Department of Clinical Molecular Oncology, Akershus University Hospital, Ahus, Norway; 55 Department of Oncology, Sheffield Cancer Research Centre, University of Sheffield, Sheffield, UK; 56 Division of Genetics and Epidemiology, Institute of Cancer Research, London, UK; 57 Division of Breast Cancer Research, Institute of Cancer Research, London, UK; 58 Department of Community and Family Medicine, Duke University School of Medicine, Durham, NC USA; 59 Division of Population Sciences, Department of Cancer Epidemiology, Moffitt Cancer Center, Tampa, FL USA

## Abstract

Several studies have reported associations between multiple cancer types and single-nucleotide polymorphisms (SNPs) on chromosome 5p15, which harbours *TERT* and *CLPTM1L*, but no such association has been reported with endometrial cancer. To evaluate the role of genetic variants at the *TERT*–*CLPTM1L* region in endometrial cancer risk, we carried out comprehensive fine-mapping analyses of genotyped and imputed SNPs using a custom Illumina iSelect array which includes dense SNP coverage of this region. We examined 396 SNPs (113 genotyped, 283 imputed) in 4,401 endometrial cancer cases and 28,758 controls. Single-SNP and forward/backward logistic regression models suggested evidence for three variants independently associated with endometrial cancer risk (*P* = 4.9 × 10^−6^ to *P* = 7.7 × 10^−5^). Only one falls into a haplotype previously associated with other cancer types (rs7705526, in *TERT* intron 1), and this SNP has been shown to alter *TERT* promoter activity. One of the novel associations (rs13174814) maps to a second region in the *TERT* promoter and the other (rs62329728) is in the promoter region of *CLPTM1L*; neither are correlated with previously reported cancer-associated SNPs. Using TCGA RNASeq data, we found significantly increased expression of both *TERT* and *CLPTM1L* in endometrial cancer tissue compared with normal tissue (*TERT* *P* = 1.5 × 10^−18^, *CLPTM1L* *P* = 1.5 × 10^−19^). Our study thus reports a novel endometrial cancer risk locus and expands the spectrum of cancer types associated with genetic variation at 5p15, further highlighting the importance of this region for cancer susceptibility.

**Electronic supplementary material:**

The online version of this article (doi:10.1007/s00439-014-1515-4) contains supplementary material, which is available to authorized users.

## Introduction

Endometrial cancer is the second most commonly diagnosed gynaecologic cancer in the world and accounts for ~5 % of all cancers in women (Kaaks et al. 2002). Worldwide, about 320,000 women are diagnosed with endometrial cancer and approximately 76,000 die of the disease annually (http://globocan.iarc.fr/Default.aspx). Risk factors for this malignancy include long reproductive span (early menarche and/or late menopause), nulliparity, obesity, hormone replacement therapy, tamoxifen, and personal and/or family history of cancer of the endometrium, breast, ovary, or colorectum (Beral et al. 2005; Fisher et al. 2005; Kaaks et al. 2002), suggesting that genetic factors play important roles in the risk of this malignancy (Hemminki et al. 2004). Endometrial cancer can be caused by rare, highly penetrant mutations in DNA repair or replication genes such as *MLH1*, *MSH2*, *MSH6*, *PMS2, POLE* or *POLD1* that result in Lynch Syndrome or in Polymerase Proofreading Associated Polyposis (Briggs and Tomlinson 2013; Fearon 1997; Palles et al. 2013). Genome-wide association studies (GWAS) have also been used to dissect the genetics of endometrial cancer and so far have convincingly identified one associated SNP, rs4430796, on chromosome 17q close to the *HNF1B* gene (Spurdle et al. 2011; Setiawan et al. 2012; Painter et al. 2014). The rs4430796 G allele is associated with decreased risks of endometrial and prostate cancers, but with an increased risk of type 2 diabetes (Gudmundsson et al. 2007). Candidate gene studies have also identified an association between endometrial cancer and two SNPs in the *CYP19A1* gene (Setiawan et al. 2009).

Variants in chromosome 5p15, a region which harbours the *TERT* and *CLPTM1L* genes, have been found through GWAS to be associated with the risk of bladder, pancreas, brain, testicular, breast, prostate, skin and lung cancers and glioma (Haiman et al. 2011; Kote-Jarai et al. 2011, 2013; McKay et al. 2008; Petersen et al. 2010; Rafnar et al. 2009; Shete et al. 2009; Stacey et al. 2009; Turnbull et al. 2010; Wang et al. 2014). *TERT* encodes the catalytic subunit of the telomerase reverse transcriptase enzyme. Activation of *TERT* transcription occurs in most human cancers where telomerase activity increases to counteract telomere shortening, thereby circumventing the normal limits on cellular proliferation (Kolquist et al. 1998). Little is known about *CLPTM1L* but recent studies have demonstrated it has an anti-apoptotic role in lung and pancreatic cancer cells (James et al. 2014; Jia et al. 2014; Wang et al. 2014). In recent studies, members of the Collaborative Oncological Gene–environment Study (COGS) used an Illumina iSelect high-density genotyping array (referred to as the “iCOGS” array) and imputation around the *TERT*–*CLPTM1L* region to identify several independent variants for breast, ovarian and prostate cancers, and for telomere length in lymphocytes (Bojesen et al. 2013; Kote-Jarai et al. 2013). In the current study, we used the iCOGS array and genotype imputation to investigate whether variants in the *TERT*–*CLPTM1L* candidate region are associated with the risk of endometrial cancer in populations of European descent.

## Materials and methods

### Samples

For the iCOGS genotyping, 5,591 women with a confirmed diagnosis of endometrial cancer and European ancestry were recruited via 11 separate studies in Western Europe, North America and Australia, collectively called the Endometrial Cancer Association Consortium (ECAC) (Supplementary Table 1). Germline DNA extracted from blood was used for genotyping.

Healthy female controls with European ancestry and known age at sampling were selected from controls genotyped by the Breast Cancer Association Consortium (BCAC) iCOGS project (Michailidou et al. 2013), or the Ovarian Cancer Association Consortium (OCAC) iCOGS project (Pharoah et al. 2013). We selected the 27,062 BCAC controls from studies in the same countries as the endometrial cancer cases, 744 European-ancestry controls from the Mayo Clinic Ovarian Cancer Case–Control Study (MAY) and 896 controls from the Australian Ovarian Cancer Study (AOCS). In addition, 282 Norwegian blood donor controls with no known history of cancer were genotyped for this study (Supplementary Table 1).

Details of cases and controls are described in the Supplementary Note.

### SNP selection and genotyping

Cases and controls were genotyped on a custom Illuminia Infinium iSelect array (“iCOGS”) with 211,155 SNPs, designed by the Collaborative Oncological Gene–environment Study, a collaborative project involving four consortia (Couch et al. 2013; Kote-Jarai et al. 2013; Michailidou et al. 2013; Pharoah et al. 2013). Cases and molecular markers in treatment of endometrial cancer (MoMaTEC) controls were genotyped by the Genome Quebec Innovation Center. BCAC and OCAC control samples were genotyped at four centres. Raw intensity data files for all consortia were sent to the COGS data coordination centre at the University of Cambridge for centralized genotype calling and QC, so that all case and control genotypes were called using the same procedure.

The study presented here relates only to SNPs within a 200 kb region (chr5:1,200,000–1,400,000) including the *TERT* and *CLPTM1L* genes. For this region, SNPs were selected for inclusion on the iCOGS array on the basis of published cancer associations and from the March 2010 release of the 1000 Genomes Project (2012). These included all known SNPs with MAF >0.02 in Europeans and *r* ^2^ > 0.1 with the then-known cancer-associated SNPs [rs402710 (McKay et al. 2008)] and/or rs3816659 (Shen et al. 2010), plus a tagging set for all known SNPs in the linkage disequilibrium blocks encompassing the genes in the region (*SLC6A18*, *TERT* and *CLPTM1L*). An additional 30 SNPs in *TERT* were selected through a telomere length candidate gene approach. In total, 134 SNPs were selected, 121 of which were successfully manufactured.

### Quality control

Genotypes were called using Illumina’s proprietary GenCall algorithm, using a cluster file specifically generated for the project using a subset of samples from each genotyping center. SNPs were excluded for call rate <95 % (<99 % for MAF <5 %), MAF <0.1 % or deviations from HWE significant at 10^−7^, based on a stratified Robinson-Hill test. Samples were excluded for low overall call rate (<95 %), heterozygosity >5 standard deviations from the mean, non-female genotype (XO, XY or XXY), or <85 % estimated European ancestry based on Identical By State scores between study individuals and individuals in HapMap (http://hapmap.ncbi.nlm.nih.gov/) and multidimensional scaling.

For duplicate samples or those identified as close relatives by IBS probabilities >0.85, the sample with the lower call rate was excluded, except for case–control relative pairs for which the case was retained. Among cases, the minimum duplicate concordance rate was 99.96 %. For cases, any 96-well plate containing ≥5 excluded samples was entirely excluded.

For 2,006 cases, we could compare iCOGS genotypes for 40 SNPs with corresponding genotypes from the rapid replication stage of our initial GWAS (Spurdle et al. 2011). Cases with unresolved discrepancies were excluded. After these exclusions, genotypes were available for 113 SNPs in the defined region, in 4,401 cases and 28,758 controls.

### Imputation

We used ImputeV2 (Howie et al. 2009) to obtain in silico genotypes for an additional 1,677 SNPs in this region using two reference panels: the 1000 Genomes Phase 1 (April 2012 release) and an in-house genotyping panel that contained 133 additional SNPs from the October 2010 1000 Genomes Project data release, genotyped in 15,044 samples from the SEARCH and CCHS BCAC studies (Bojesen et al. 2013). After filtering for SNP frequency (MAF ≥0.02; 887 SNPs excluded) and imputation QC (info score ≥0.8; 394 further SNPs excluded), we included 396 SNPs in the association analyses, comprising 113 genotyped and 283 imputed. SNPs with MAF <0.02 were excluded because we would not have statistical power to detect associations with rare SNPs. We used a stringent cutoff for the imputation information score to reduce the chance of spurious associations caused by imputation artefacts. The IMPUTEv2 “leave-out” internal concordance check gave 98.2 % concordance at SNPs with *r* ^2^ ≥ 0.8 for SNPs on the 1000 Genomes reference panel but not on the additional in-house panel, and 99.2 % for those SNPs also on the in-house reference panel.

### Statistical analysis

Associations between each SNP and endometrial cancer were estimated using unconditional logistic regression with a per-allele (1df) model, based on the expected genotype dosages for the imputed SNPs. Analyses were adjusted for strata (6 of the 8 strata were defined by country, whilst the large UK dataset was divided into ‘SEARCH’ and ‘other UK’) and for the first 10 principal components of the genomic kinship matrix, based on 37,000 uncorrelated SNPs (*r* ^2^ < 0.1), including ~1,000 selected as ancestry informative markers, using an in-house C++ programme incorporating the Intel MKL libraries for eigenvectors (http://ccge.medschl.cam.ac.uk/software/). One principal component was derived specifically for the Leuven (LES/LMBC) studies, for which there was substantial inflation not accounted for by the other principal components.

Inflation of the test statistic (*λ*) was estimated by dividing the 45th centile of the test statistic by the 45th centile of a 1*df* *χ* ^2^ distribution based on 43,233 uncorrelated (*r* ^2^ < 0.1) SNPs selected for the iCOGS array by consortia other than ECAC. This was converted to an equivalent inflation for a study with 1,000 cases and 1,000 controls (*λ*_1,000_) by adjusting for effective sample size,

$$\lambda_{1,000} = 1 + \frac{{500\left( {\lambda - 1} \right)}}{{\sum\nolimits_{k} {\left( {\frac{1}{{{\text{ncase}}_{k} }} + \frac{1}{{{\text{nctrl}}_{k} }}} \right)} }}$$

where ncase_*k*_ and nctrl_*k*_ are the numbers of cases and controls in strata *k*.

A ‘global’ test using the admixture maximum likelihood method [AML (Tyrer et al. 2006)] was performed against the null hypothesis that none of the genotyped SNPs within the region are associated with endometrial cancer, with the alternative hypothesis that at least one of the SNPs is associated, based on 10,000 permutations. The test was performed for 55 of the 113 genotyped SNPs, selected such that none of the SNPs had a pairwise *r* ^2^ ≥ 0.5 with another SNP in the test.

To determine independently associated SNPs, we used forward stepwise logistic regression based on all SNPs with *P* < 0.05 in the single-SNP analysis; at each stage, the most significant SNP was potentially eligible for inclusion in the final model if it was significant at *P* < 0.01 after adjustment for other SNPs. Given the strong prior evidence of cancer associations with this region, this is a candidate gene study, and hence the very stringent significance thresholds required for a GWAS are not applicable here. The 396 SNPs in the analysis can be pairwise-tagged by 68 tagging SNPs at *r* ^2^ ≥ 0.5, hence the number of strictly independent tests is closer to 68 than to 396 (and could be considered to be even lower) which would give a Bonferroni-corrected significance threshold of around 0.05/68 = 7.4 × 10^−4^. An additional logistic regression was performed including all SNPs retained in the step-wise process. Backwards logistic regression was also performed. A secondary analysis was performed in which the most significant independent SNPs from the main analysis were tested for associations specifically with endometrioid and non-endometrioid histology endometrial cancer, and in a case-only comparison of endometrioid and non-endometrioid cases. Pairwise linkage disequilibrium *r* ^2^ measures were calculated from the iCOGS samples.

As an alternative to the frequentist stepwise variable selection procedure we also used a Bayesian-inspired penalized maximum likelihood approach which simultaneously analyses all genotyped and imputed SNPs in the region to identify the optimal subset for disease prediction [HyperLasso (Hoggart et al. 2008)]. We used the normal exponential gamma distribution (NEG) shrinkage prior with shape parameter 1.0, as recommended by Vignal et al. (2011). To obtain a SNP-wise type I error of 0.001, we used a penalty (lambda) of 110, estimated based on 100 permutations under the null for different values of lambdas.

The Tagger package (de Bakker et al. 2005) was used to identify independent tagging SNPs for the AML analysis. All analyses were conducted using R, including the GenABEL and SNPMatrix packages (Aulchenko et al. 2007; Clayton and Leung 2007), apart from the HyperLasso analysis (Hoggart et al. 2008) and the AML testing (Tyrer et al. 2006). All statistical tests were 2-sided.

### SNP annotation

We annotated all SNPs that had moderate to high LD with the three risk alleles identified in our study using Galaxy (Giardine et al. 2005) and the UCSC genome browser. To do so, we followed the annotation scheme described recently by Carvajal-Carmona et al. (2011).

### Gene expression analysis

A literature search to identify all published microarray studies investigating endometrial cancer was performed and datasets accessed directly from the author (Moreno-Bueno et al. 2003), publication supplementary data (Risinger et al. 2003; Saidi et al. 2004) or the NCBI Gene Expression Omnibus database [GEO; http://www.ncbi.nlm.nih.gov/geo/; (Day et al. 2011) (GSE17025), (Mhawech-Fauceglia et al. 2010) (GSE23518), (Salvesen et al. 2009) (GSE14860)]. Additional microarray data were downloaded from the Expression Project for Oncology (expO) study via GEO (GSE2190) and TCGA (Kandoth et al. 2013) via the TCGA data portal (http://tcga-data.nci.nih.gov/tcga/tcgaHome2.jsp). *TERT* expression was interrogated by the platforms used in all eight datasets, whilst *CLPTM1L* was able to be interrogated by five datasets [(Day et al. 2011; Kandoth et al. 2013; Mhawech-Fauceglia et al. 2010; Salvesen et al. 2009) and expO].

All datasets were log transformed (by taking the logarithmic values of the signals to the base of two) and median centred per array. The change in expression level of *TERT* and *CLPTM1L* between non-endometrioid and endometrioid endometrial cancer for each individual study was expressed as an effect size, a unit-free standardized mean difference between groups. Gene expression results were then combined using the t-based modelling approach (Choi et al. 2003) using the meta-package in R. Meta-analysis was performed using a random effects model to account for between-study heterogeneity.

Level 3 (processed) RNASeqV2 normalized expression values for TCGA endometrial cancer samples were downloaded from the TCGA data portal. Differences in *TERT* and *CLPTM1L* expression between cancer vs normal tissue and endometrioid vs non-endometrioid endometrial cancer tissue was assessed by Mann–Whitney *U* test using IBM SPSS Statistics (version 22).

### eQTL analysis

Level 2 (preprocessed) germline GWAS data from endometrial cancer patients was downloaded from the TCGA data portal and QC performed. SNPs were excluded for call rate <95 %, MAF <1 % or deviations from HWE significant at 10^−4^. Samples were excluded for low overall call rate (<95 %), heterozygosity >3 standard deviations from the mean, inconclusive sex status (X-chromosome homozygosity rate between 0.2 and 0.8), or samples >6 standard deviations from the mean scores for principal component 1 or 2, calculated using CEU individuals in HapMap (http://hapmap.ncbi.nlm.nih.gov/). For duplicate samples or samples identified as close relatives by IBS probabilities >0.85, the sample with the lower call rate was excluded. RNA-Seq Zscores and GISTIC copy number calls for TCGA endometrial cancer samples were obtained via the cBio Portal for Cancer Genomics (http://www.cbioportal.org/public-portal/index.do). There were 192 TCGA samples with both genotype and gene expression data available for analysis. The association of SNPs in the *TERT*–*CLPTM1L* gene region (chr5:1,200,000–1,400,000) with *TERT* and *CLPTM1* expression was assessed using PLINK, adjusting for copy number.

## Results

We performed high-density genotyping and genotype imputation for variants in the 5p15 *TERT*–*CLPTM1L* region to examine genetic associations with endometrial cancer risk. For this purpose, we used a custom-designed Illumina iSelect ~200,000 SNP array (“iCOGS”), which included 118 successfully genotyped SNPs (after standard QC exclusions) spanning a 200 kb region (chr5:1,200,000–1,400,000), to genotype 4,401 endometrial cancer cases from 11 centres participating in the Endometrial Cancer Association Consortium (ECAC) and 28,758 control subjects from the Breast Cancer Association Consortium (BCAC) and the Ovarian Cancer Association Consortium (OCAC). All subjects were of European ancestry (Supplementary Table 1). We then imputed the genotypes of untyped SNPs using 1000 Genomes project data (April 2012 release) as a reference. After excluding SNPs with an imputation information score <0.8 or minor allele frequency <0.02, 113 genotyped and 283 imputed SNPs were included in the analyses. There was no evidence of genomic inflation (*λ*_1,000_ = 1.012, based on 43,233 uncorrelated iCOGS SNPs separate from those presented here).

First, a ‘global’ test using the admixture maximum likelihood method (AML) (Tyrer et al. 2006) against the null hypothesis that none of the genotyped SNPs within the *TERT*–*CLPTM1L* region are associated with endometrial cancer provided significant evidence that at least one SNP is associated (*P* = 0.0001).

**Fig. 1:**
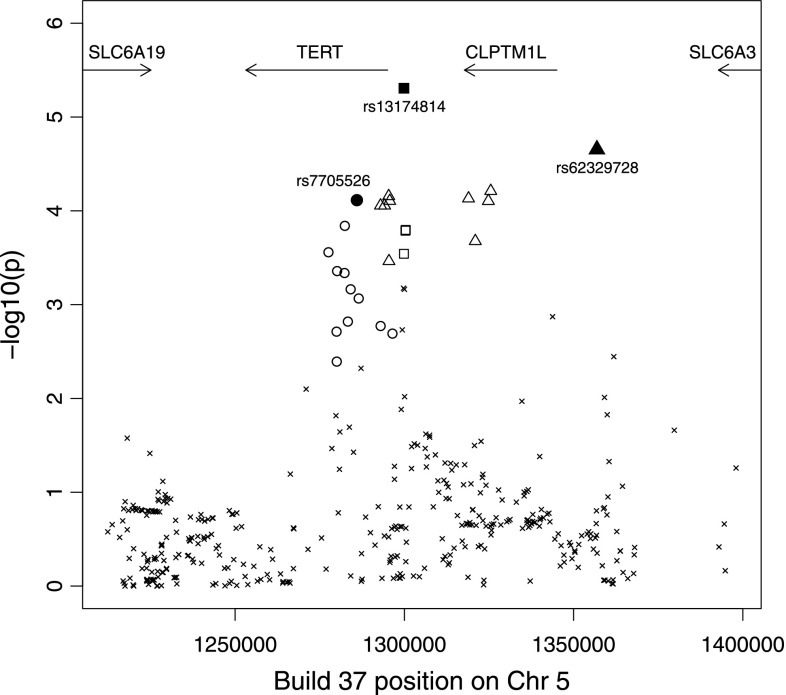
Association between SNPs in the 5p15 region and endometrial cancer. SNPs in SNP sets 1–3 are shown by *circles*, *squares* and *triangles*, respectively, with the filled symbols denoting the most significant SNP in that set. Only SNPs with MAF >0.02 and imputation information score >0.8 are shown

Single-SNP association testing identified 61 out of 396 SNPs with *P* values <0.05, compared with <20 expected by chance (Fig. 1; Supplementary Table 2). Forward stepwise logistic regression based on these 61 SNPs identified three imputed SNPs (rs7705526, rs13174814 and rs62329728) that each showed evidence of being independently associated with disease (*P* = 7.7 × 10^−5^, 4.9 × 10^−6^ and 2.2 × 10^−5^; conditioning on the other SNPs in the model *P* = 9.7 × 10^−3^, 1.7 × 10^−4^ and 1.8 × 10^−4^, respectively; Table 1). The three SNPs had high imputation information scores (0.89, 0.98 and 0.82, respectively). Backward stepwise regression did not improve the model. The linkage disequilibrium (LD) between these three SNPs is weak (maximum pairwise *r* ^2^ = 0.047; Table 1), which further suggests that they represent independent risk factors for endometrial cancer. Although rs7705526 did not reach the approximate Bonferroni-corrected significance threshold (7.4 × 10^−4^; see “Materials and methods”), it was retained in the model because of its individual significance and the strong prior evidence supporting a role for this particular SNP in hormonal cancers (Bojesen et al. 2013).

**Table 1:** The 3 SNPs showing independent associations with endometrial cancer

SNP	Position (bld 37)	A1/A2	Frequency of A1	Imputation information score	Unconditional analysis		Conditional analysis		*r* ^2^ with rs7705526	*r* ^2^ with rs13174814
					OR (95 % CI)	*P* value	OR (95 % CI)	*P* value		
rs7705526	1,285,974	C/A	0.33	0.89	1.11 (1.06, 1.17)	7.7E−05	1.08 (1.02, 1.14)	9.7E−03		
rs13174814	1,299,859	G/C	0.25	0.98	0.87 (0.82, 0.93)	4.9E−06	0.89 (0.84, 0.95)	1.7E−04	0.047	
rs62329728	1,356,890	G/A	0.06	0.82	1.27 (1.14, 1.43)	2.2E−05	1.24 (1.11, 1.39)	1.8E−04	0.024	<0.001

Unconditional analyses were adjusted for study strata (see “Materials and methods”) and for the first ten principal components. The Conditional Analysis model was also adjusted for the above variables and contained all 3 listed SNPs. Odds ratios (OR) are for allele A1

Whilst the three SNPs in Table 1 were the most significant in the forward logistic regression, each SNP should be considered as a tagging or representative SNP for a set of SNPs, sometimes referred to as an association “peak”. For each of the three SNPs, Supplementary Table 3 lists all other SNPs in the analysis which were in LD (*r* ^2^ > 0.2) with that SNP, and which have likelihood ratios of <100:1 relative to the most significant SNP for that set. The SNP sets harbouring rs7705526, rs13174814 and rs62329728 (SNP sets 1, 2 and 3), respectively, contain 12, 4 and 10 distinct SNPs, none of which could be excluded as potentially causative on the basis of statistical analysis. Replacing each of the three imputed SNPs in Table 1 with a genotyped SNP from its own SNP set, each SNP set still showed evidence of association with endometrial cancer in the multi-SNP model, albeit with slightly weaker significance for two of the three sets, indicating that the observed effects are not due to imputation artefacts (Supplementary Table 4).

As an alternative to the frequentist stepwise variable selection procedure, we also used a Bayesian-inspired penalized maximum likelihood approach which simultaneously analyses all genotyped and imputed SNPs in the region to identify the optimal subset for disease prediction [HyperLasso (Hoggart et al. 2008)]. With shrinkage parameters fixed to obtain a Type I Error Rate of 0.001, the four best-fitting models all contained rs13174818 (lead SNP in SNP set 2), and one of rs7705526, rs33961405, rs7725218 or rs7734992, all of which fall within SNP set 1. This differs in some respects from the stepwise regression results, in which rs13174814 and rs62329728 were more significant than rs7705526, and provides further support for a role of SNP set 1 in endometrial cancer.

Of the three SNPs independently associated with endometrial cancer in our study, only one (rs7705526) lies in an LD region previously associated with cancer risk. rs7705526 (OR = 1.11, CI = 1.06–1.17, *P* = 7.7 × 10^−5^) is located in the first intron of *TERT* (chr5:1,285,974, Supplementary Fig. 1a). In the recent COGS study of breast and ovarian cancer risk and telomere length associated with SNPs in the *TERT* region, rs7705526 was classified as being in what was referred to as “peak 2” (one of two sets of associated SNPs straddling *TERT* introns 2–4 in that study), and was associated with longer telomeres in blood cells and with increased risks of breast cancer (oestrogen receptor negative and positive subtypes) and ovarian cancer (serous low-malignant potential and serous invasive epithelial) (Bojesen et al. 2013; Pharoah et al. 2013). rs7705526 is in high LD with prostate cancer SNP rs7725218 (*r* ^2^ = 0.87) (Kote-Jarai et al. 2013), and also in moderate LD with SNPs in “peak 3” of the COGS study, e.g., *r* ^2^ = 0.36 with rs10069690, which is particularly associated with oestrogen receptor negative breast cancer and with both subtypes of ovarian cancer (Supplementary Table 5) (Bojesen et al. 2013; Pharoah et al. 2013). rs7705526 is also in LD with rs7726159 and rs2736100 (*r* ^2^ = 0.95 and 0.53, respectively, Supplementary Table 5), which are reported to be associated with multiple cancers including lung, ovarian, testicular, pancreatic and prostate cancers and glioma. Therefore, rs7705526 lies in a complex risk haplotype that is now associated with risks of at least eight different types of cancers.

The two remaining SNP sets identified as independently associated with endometrial cancer risk in our study (represented by rs13174814 and rs62329728) have not, to the best of our knowledge, been previously associated with cancer (Supplementary Table 5), and therefore represent novel risk variants in the region. rs13174814 (OR = 0.87, CI = 0.82–0.93, *P* = 4.9 × 10^−6^) maps to the *TERT* promoter (chr5: 1,299,859 and ~4.7 Kb from the 5′ UTR), a region that has been previously associated with the risk of testicular [rs4635969 (Turnbull et al. 2010)], lung [rs4975616 (Landi et al. 2009; Wang et al. 2008)], prostate [rs7712562, rs2853669, rs2736107 and rs13190087 (Kote-Jarai et al. 2013)] and breast cancers [rs2853669, rs2736108 and rs2736107 (Bojesen et al. 2013)]. However, the previously reported cancer-associated variants show only weak LD with rs13174814 (*r* ^2^ < 0.07 for all comparisons) (Supplementary Table 5), suggesting that this SNP represents a novel risk variant for cancer in the promoter region of *TERT*. The other SNP independently associated with endometrial cancer, rs62329728 (OR = 1.27, CI = 1.14–1.43, *P* = 2.2 × 10^−5^), maps to a non-coding region ~12 kb upstream of the 5′ UTR of *CLPTM1L* (Supplementary Fig. 1c). To the best of our knowledge, rs62329728 is not correlated with any published cancer SNP (*r* ^2^ < 0.05), and thus represents a new cancer risk allele in the *CLPTM1L* region.

rs13174814 and rs62329728 showed similar associations for endometrioid and the more aggressive non-endometrioid histology endometrial cancers (Supplementary Table 6). Although rs7705526 was not significantly associated with non-endometrioid cancers, the number of non-endometrioid cancers (*n* = 757) was far smaller than the number of endometrioid cancers (*n* = 3,535), and the case-only endometrioid vs non-endometrioid analyses did not show any significant differences (*P* > 0.05).

**Fig. 2:**
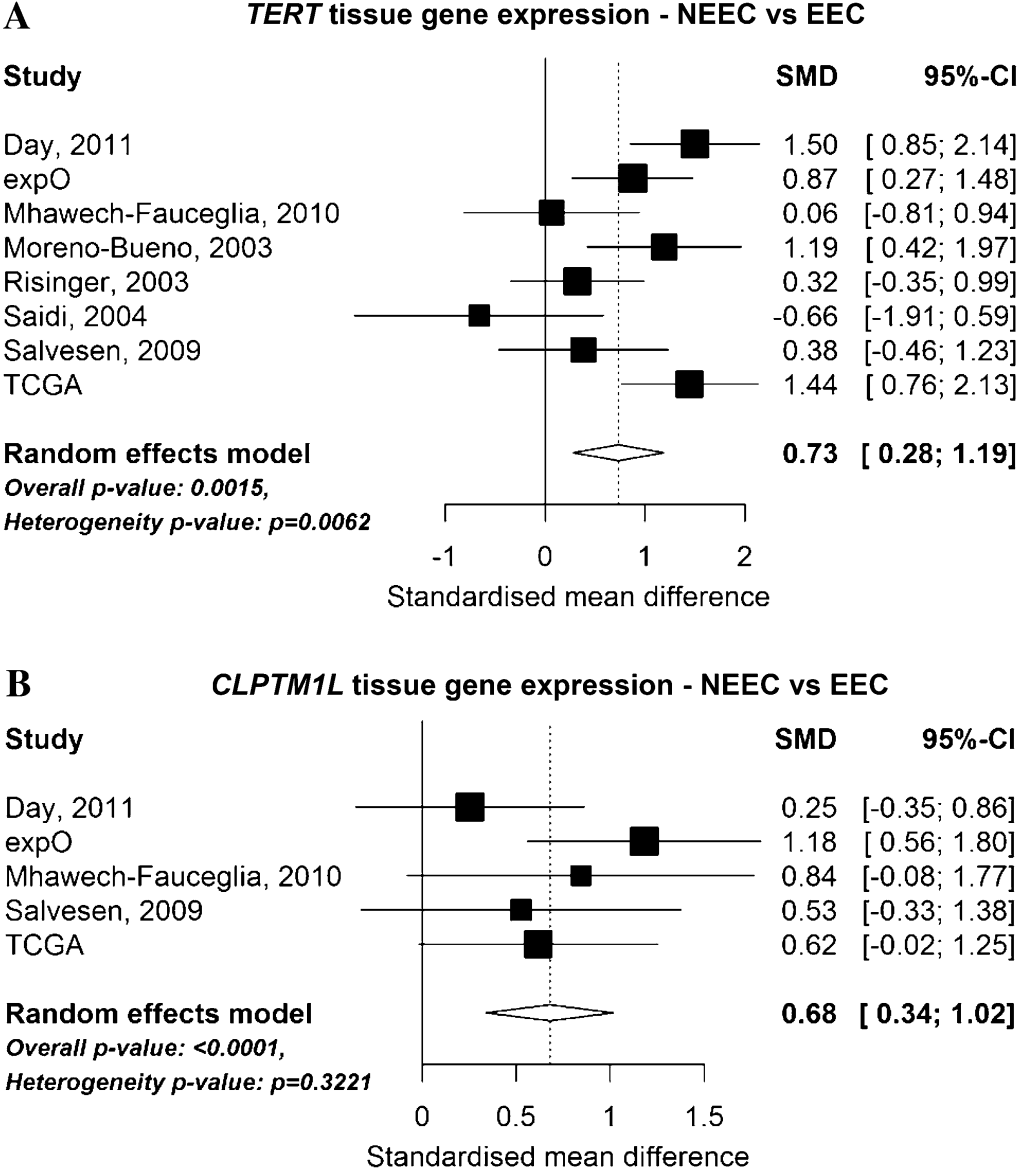
Forest plot showing the differential expression of **a** *TERT* and **b** *CLPTM1L* by endometrial cancer histological subtype using collated datasets of endometrial cancer microarray gene expression. The *solid vertical line* represents no change in gene expression between the two histological subtypes and the *dashed line* indicates the overall standardized mean difference (SMD) in expression across all studies analysed. SMD is a unit-free measurement of gene expression. A positive SMD value represents increased gene expression in non-endometrioid endometrial cancer (NEEC) compared with endometrioid endometrial cancer (EEC). Heterogeneity *P* value was calculated by Q-statistic

**Fig. 3:**
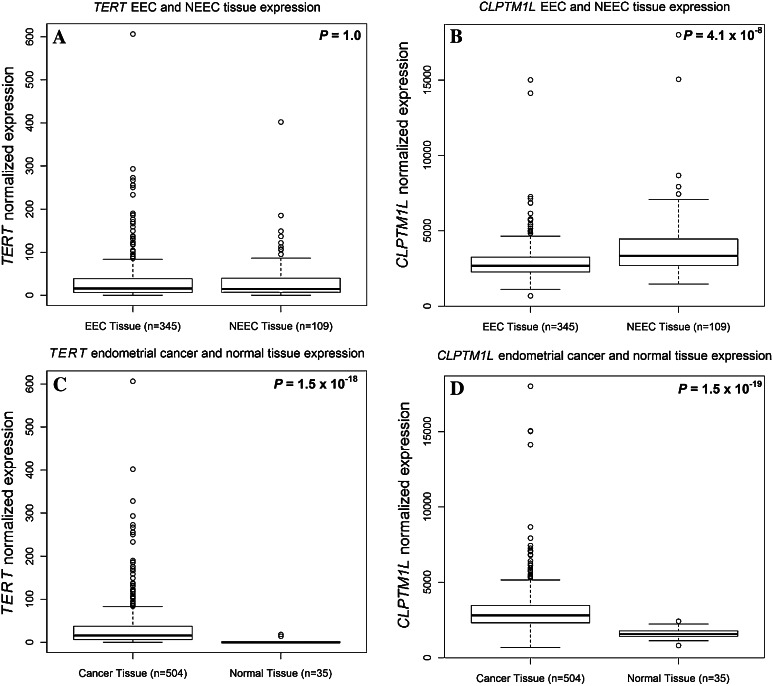
*Boxplots* of endometrial tissue normalized gene expression levels using RNASeq data generated by The Cancer Genome Atlas. *Boxplots* depict the median and first and third quartiles. **a** *TERT* expression in endometrioid endometrial cancer (EEC) and non-endometrioid endometrial cancer (NEEC) tissue samples. **b** *CLPTM1L* expression in EEC and NEEC tissue samples. **c** *TERT* expression in endometrial cancer and normal endometrial tissue. **d** *CLPTM1L* expression in endometrial cancer and normal endometrial tissue

To identify possible mechanistic associations between *TERT, CLPTM1L* and endometrial cancer, we searched for information on endometrial gene expression and somatic variation in publically available datasets. Specifically, we looked at eight microarray datasets that have compared gene expression levels in endometrioid and non-endometrioid cancer (Fig. 2) and RNASeq data from The Cancer Genome Atlas (TCGA, Fig. 3). Analysis of microarray data found that *TERT* was overexpressed in non-endometrioid cancer (*P* = 0.0015, Fig. 2a), however, this was not observed in the larger TCGA RNASeq dataset (*P* = 1.0, Fig. 3a). Increased expression of *CLPTM1L* in non-endometrioid cancer was seen across five of the microarray datasets that also interrogated *CLPTM1L* expression (*P* < 0.0001, Fig. 2b), with a similar result also found by the TCGA RNASeq analysis (*P* = 4.1 × 10^−8^, Fig. 3b). Using TCGA RNASeq data we found significantly increased expression of both *TERT* (Fig. 3c) and *CLPTM1L* (Fig. 3d) in endometrial cancer tissue compared with normal tissue (*TERT* *P* = 1.5 × 10^−18^, *CLPTM1L* *P* = 1.5 × 10^−19^). TCGA endometrial cancer data analysis (http://www.cbioportal.org/public-portal/index.do) shows that the 5p15.33 region containing both *TERT* and *CLPTM1L* is significantly amplified in ~3 % of cases (Gistic *Q* value <0.00011, not shown), whilst *TERT* and *CLPTM1L* mutations have been identified in a small fraction of endometrial tumours (Kandoth et al. 2013).

We then assessed association between SNPs in the region and *TERT* and *CLPTM1L* expression. Our most strongly associated risk variants were not genotyped by the TCGA genotyping platform (Affymetrix 6.0) and it was not possible to impute these SNPs with a satisfactory degree of accuracy (imputation information scores of 0.41, 0.35 and 0.45 for rs7705526, rs13174814 and rs62329728, respectively) based on this genotyping. Other variants in the region were assessed for association with expression of *TERT* (Supplementary Table 7) or *CLPTM1L* (Supplementary Table 8): the best *TERT* eQTL (*P* = 0.009) was for rs2853668 (endometrial cancer risk *P* = 7.2 × 10^−4^; Supplementary Table 2) located 166 bp from rs13174814 (*r* ^2^ = 0.10) in the *TERT* promoter; the best *CLPTM1L* eQTL (*P* = 0.06) was observed for rs2736100 (endometrial cancer risk *P* = 8.6 × 10^−4^; Supplementary Table 2), located 542 bp from rs7705526 (*r* ^2^ = 0.53). The TCGA genotyping array provided reasonable tags for rs7705526 (best tag rs2736100 with *r* ^2^ = 0.53), but not for rs62329728 (best tag rs246992, *r* ^2^ = 0.09) or rs1317814 (best tag rs246995, *r* ^2^ = 0.13).

## Discussion

Using high-density genotyping, imputation, a ‘global’ likelihood test and multi-SNP logistic regression analyses, we have shown for the first time that genetic variants in the *TERT*–*CLPTM1L* region are associated with the risk of endometrial cancer, and provide evidence that this region contains three independent risk SNPs for this cancer. One previous study has reported a nominally significant association between a SNP in the *TERT* region (rs2736122) and endometrial cancer (reported *P* = 0.03) (Prescott et al. 2010), but this SNP was not significant in our larger analysis (*P* = 0.85; Supplementary Table 5), whilst a recent multi-cancer study of nearly 2,000 5p15.33 SNPs did not report an association with endometrial cancer (Wang et al. 2014). Only one of the endometrial cancer risk variants identified in our study (rs7705526) lies in an LD region that has been previously associated with other cancer types.

To date, GWAS for endometrial cancer have convincingly identified evidence for endometrial cancer risk association at the *HNF1B* locus (Spurdle et al. 2011; Setiawan et al. 2012; Painter et al. 2014), the risk allele of which (rs4430796A) maps to a region that has also been associated with the risk of ovarian and prostate cancers (Gudmundsson et al. 2007; Shen et al. 2013; Thomas et al. 2008). In the candidate study of the 5p15 multi-cancer region presented here, we have identified up to three new independent endometrial cancer risk variants within a locus already associated with multiple cancers, potentially accounting for ~0.5 % of the excess familial relative risk of endometrial cancer. A similar candidate region approach has been used successfully to demonstrate associations between variation at the 8q24 multi-cancer region and thyroid cancer, another understudied malignancy (Jones et al. 2012). We thus propose that future studies on the role of additional multi-cancer regions, such as 1q32/*MDM4*, 4q24/*TET2*, 8q24, 10p12/*MLT10*, 14q24/*RAD51B8* or 19q13/*MERIT40* (Sakoda et al. 2013), are worthwhile endeavours for cancers that are relatively understudied, including endometrial cancer.

Among the list of 41 *TERT* SNPs for which we were able to identify a previous report of a significant association with cancer in a European ancestry population (Supplementary Table 5), only those SNPs which are in LD with rs7705526 showed even nominally significant associations with endometrial cancer (with the exceptions of *P* = 0.032 for rs402710 and *P* = 0.041 for rs13172201), and none remained significant after conditioning on rs7705526. This suggests that we identified one SNP from a haplotype which is associated with endometrial cancer and also with multiple other types of cancer, and two mutually independent SNPs which are associated with endometrial cancer but do not lie in haplotypes previously reported to be associated with any other type of cancer. However, this does not exclude the possibility that these novel endometrial cancer SNPs are also multi-cancer variants. The 5p15.33 region has complex LD patterns and is poorly tagged by many GWAS genotyping panels. As a comparison, we examined the SNP coverage of this region in a set of 5,180 control subjects genotyped using the Illumina Infinium 1.2M GWAS array as part of the Wellcome Trust Case Control Consortium (2007), for which missing genotypes were imputed using the same method and reference panel as in our main study. Of the 799 SNPs with MAF >0.02, the median imputation information score in the iCOGS set was 0.80 compared with 0.21 in the 1.2M GWAS set, and 87 % of SNPs had an information score of at least 0.4 in the iCOGS set compared to just 26 % of SNPs reaching this threshold in the GWAS set (Supplementary Fig. 2; Supplementary Table 2). These findings emphasize the value of targeted, dense genotyping as a complementary approach to standard GWAS. The imputation information score for rs7705526 (the only one of our associated SNPs previously associated with other cancer types) was 0.55 in the GWAS set, whilst the GWAS information scores for rs13174814 and rs62329728 were just 0.43 and 0.12, respectively. Thus, the use of a deliberately dense panel of local SNPs, such as that used in this study, may reveal associations between the novel endometrial cancer risk SNPs and other cancers.

Fine-mapping genomic regions which potentially contain multiple causal variants is a relatively new area of research, and generally accepted thresholds for claiming the statistical significance of variants do not yet exist. An appropriate threshold for a given region can depend on the number of SNPs tested, the extent of LD in the region, the frequencies of the variants and the prior evidence for association. Some authors have suggested using Bayesian inference as an alternative to frequentist *P* value-based methods. Here, we performed one such Bayesian-inspired method, the HyperLasso (Hoggart et al. 2008), which also found associations with SNP sets 1 and 2, but reported no further associated SNPs. The results of this alternative method increase our confidence in the associations between endometrial cancer and SNP sets 1 and 2, while direct genotyping of large case–control studies will help towards resolving the disagreement between statistical methods regarding the associations with SNP set 3. The use of imputed genotypes in our analysis allowed us to examine a broader group of SNPs than would have been possible in an analysis restricted to SNPs that had been genotyped. Genotyping cases and controls using the same array, thorough pre-imputation quality control, excluding rarer SNPs and restricting the analysis to SNPs with high imputation information scores (>0.8) should have reduced imputation errors and minimized the chance of false-positive associations (Marchini and Howie 2010). Nevertheless, it will be informative to replicate the analysis using direct genotyping in independent samples.

Two of the endometrial cancer risk SNPs identified in this study are in or near the *TERT* gene. The risk allele at rs7705526 has been shown to result in increased *TERT* promoter activity in luciferase reporter assays conducted in ER-negative breast, ER-positive breast and ovarian cancer cell lines (Bojesen et al. 2013), and was reported to be associated with *TERT* transcript levels in benign prostate tissue (Kote-Jarai et al. 2013). Data from ENCODE show that rs13174814 and another SNP in LD with it, rs13174919, map to a 400 bp region (chr5:1,299,601–1,300,000) identified as an insulator in embryonic stem cells, although an insulator function has yet to be experimentally validated in this or other cell lines. Interestingly, there are also a number of chromatin interactions, indicative of regulatory potential in the region of the most likely causal SNPs for this SNP set in two cancer cell lines (MCF7 and K562) (Supplementary Fig. 1b). Furthermore, our search for functional effects in RegulomeDB (Boyle et al. 2012) and HaploReg (Ward and Kellis 2012) suggests that rs13174814 affects the binding of both RAD21 and CTCF. Previous studies have shown that both RAD21 and CTCF are deregulated or aberrantly expressed in endometrial cancer (Hoivik et al. 2014; Supernat et al. 2012). Interestingly, CTCF appears to be a target for slippage mutations in endometrial cancers with microsatellite instability (Zighelboim et al. 2014).

The third endometrial cancer risk SNP identified in this study is in the upstream/promoter region of *CLPTM1L*, ~60 kb away from *TERT*, and which also harbours several cancer risk alleles, mostly for non-hormone-related malignancies such as lung, bladder and pancreatic cancers (Haiman et al. 2011; Kote-Jarai et al. 2011, 2013; McKay et al. 2008; Petersen et al. 2010; Rafnar et al. 2009; Shete et al. 2009; Stacey et al. 2009; Turnbull et al. 2010; Wang et al. 2014). The evidence for an involvement of *CLPTM1L* in tumorigenesis is, however, more limited. One study has linked *CLPTM1L* expression with cisplatin resistance in an ovarian cancer cell line (Yamamoto et al. 2001) and more recently, *CLPTM1L* was shown to promote growth and enhance chromosomal instability in pancreatic cancer cell lines (Jia et al. 2014). Although yet to be functionally characterized, rs62329728 is in LD (*r* ^2^ > 0.8) with additional SNPs across the *TERT*–*CLPTM1L* region which are located within areas of open chromatin, transcription factor binding or chromatin interactions in multiple ENCODE cell lines including the Ishikawa endometrial cancer cell line (Supplementary Fig. 1c), and hence may have regulatory potential.

Our analysis of microarray datasets suggested differences in *CLPTM1L* expression between endometrial tumour histological subtypes, and increased expression of both *TERT* and *CLPTM1L* between endometrial tumour and normal tissue. Further, a role for *TERT* is indicated by eQTL analyses, in that endometrial cancer risk-associated SNPs were associated with expression of *TERT* in endometrial tumour tissue. These results have highlighted a new region of the *TERT* promoter worthy of functional investigation, and, importantly, implicate *CLPTM1L* expression in the aetiology of endometrial cancer. As such, these findings will expand biological studies of the *TERT*/*CLPTM1L* region in this and other hormone-driven cancers. A possibility that should be examined in future studies is the existence of long-range regulatory elements in this region and their effects on *TERT*, and whether the prioritized risk-associated variants play a role in *CLPTM1L* regulation.

In summary, we have used an informed candidate approach to identify a novel endometrial cancer risk locus. Importantly, our study highlights the value of using the information generated by GWAS to guide candidate gene/SNP approaches, particularly for those cancer types that have been relatively understudied using the GWAS approach, such as endometrial cancer. Unlike previous studies in hormone-related malignancies (breast, ovarian and prostate), which only found risk variants in or near *TERT*, our study found evidence of risk variants in and near *TERT* and also near *CLPTM1L*. Future studies should investigate the functional effects of prioritized risk-associated variants on *CLPTM1L* and/or *TERT* in endometrial cancer and other cancer models. Furthermore, additional studies, ideally using re-sequencing, should be carried out to uncover possible additional low frequency causal variants.

## Electronic supplementary material

Below is the link to the electronic supplementary material.

Supplementary material 1 (PDF 259 kb)

Supplementary Fig. 2: Histograms comparing the SNP coverage of the chromosome 5 1,200,000–1,400,000 region by the Illuminia Infinium 1.2 M GWAS array (as genotyped in 5180 control subjects from the Wellcome Trust Case Control Consortium) with that of the Illuminia iSelect iCOGS array genotyped in this study, as captured by the quality of imputation to the 1000 Genomes April 2012 reference panel (PDF 4 kb)

Supplementary material 3 (XLS 2200 kb)

Supplementary material 4 (DOC 124 kb)

## References

[CR1] Aulchenko YS, Ripke S, Isaacs A, van Duijn CM (2007). GenABEL: an R library for genome-wide association analysis. Bioinformatics.

[CR2] Beral V, Bull D, Reeves G (2005). Endometrial cancer and hormone-replacement therapy in the Million Women Study. Lancet.

[CR3] Bojesen SE, Pooley KA, Johnatty SE, Beesley J, Michailidou K, Tyrer JP, Edwards SL, Pickett HA, Shen HC, Smart CE et al (2013) Multiple independent variants at the TERT locus are associated with telomere length and risks of breast and ovarian cancer. Nat Genet 45:371–384, 384e371–372. doi:10.1038/ng.2566 10.1038/ng.2566 PMC3670748 23535731

[CR4] Boyle AP, Hong EL, Hariharan M, Cheng Y, Schaub MA, Kasowski M, Karczewski KJ, Park J, Hitz BC, Weng S (2012). Annotation of functional variation in personal genomes using RegulomeDB. Genome Res.

[CR5] Briggs S, Tomlinson I (2013). Germline and somatic polymerase epsilon and delta mutations define a new class of hypermutated colorectal and endometrial cancers. J Pathol.

[CR6] Carvajal-Carmona LG, Cazier JB, Jones AM, Howarth K, Broderick P, Pittman A, Dobbins S, Tenesa A, Farrington S, Prendergast J (2011). Fine-mapping of colorectal cancer susceptibility loci at 8q23.3, 16q22.1 and 19q13.11: refinement of association signals and use of in silico analysis to suggest functional variation and unexpected candidate target genes. Hum Mol Genet.

[CR7] Choi JK, Yu U, Kim S, Yoo OJ (2003). Combining multiple microarray studies and modeling interstudy variation. Bioinformatics.

[CR8] Clayton D, Leung HT (2007). An R package for analysis of whole-genome association studies. Hum Hered.

[CR9] Couch FJ, Wang X, McGuffog L, Lee A, Olswold C, Kuchenbaecker KB, Soucy P, Fredericksen Z, Barrowdale D, Dennis J (2013). Genome-wide association study in BRCA1 mutation carriers identifies novel loci associated with breast and ovarian cancer risk. PLoS Genet.

[CR10] Day RS, McDade KK, Chandran UR, Lisovich A, Conrads TP, Hood BL, Kolli VS, Kirchner D, Litzi T, Maxwell GL (2011). Identifier mapping performance for integrating transcriptomics and proteomics experimental results. BMC Bioinform.

[CR11] de Bakker PI, Yelensky R, Pe’er I, Gabriel SB, Daly MJ, Altshuler D (2005). Efficiency and power in genetic association studies. Nat Genet.

[CR12] Fearon ER (1997). Human cancer syndromes: clues to the origin and nature of cancer. Science.

[CR13] Fisher B, Costantino JP, Wickerham DL, Cecchini RS, Cronin WM, Robidoux A, Bevers TB, Kavanah MT, Atkins JN, Margolese RG (2005). Tamoxifen for the prevention of breast cancer: current status of the National Surgical Adjuvant Breast and Bowel Project P-1 study. J Natl Cancer Inst.

[CR14] Genomes Project C, Abecasis GR, Auton A, Brooks LD, DePristo MA, Durbin RM, Handsaker RE, Kang HM, Marth GT, McVean GA (2012). An integrated map of genetic variation from 1,092 human genomes. Nature.

[CR15] Giardine B, Riemer C, Hardison RC, Burhans R, Elnitski L, Shah P, Zhang Y, Blankenberg D, Albert I, Taylor J (2005). Galaxy: a platform for interactive large-scale genome analysis. Genome Res.

[CR16] Gudmundsson J, Sulem P, Steinthorsdottir V, Bergthorsson JT, Thorleifsson G, Manolescu A, Rafnar T, Gudbjartsson D, Agnarsson BA, Baker A (2007). Two variants on chromosome 17 confer prostate cancer risk, and the one in TCF2 protects against type 2 diabetes. Nat Genet.

[CR17] Haiman CA, Chen GK, Vachon CM, Canzian F, Dunning A, Millikan RC, Wang X, Ademuyiwa F, Ahmed S, Ambrosone CB (2011). A common variant at the *TERT–CLPTM1L* locus is associated with estrogen receptor-negative breast cancer. Nat Genet.

[CR18] Hemminki K, Rawal R, Chen B, Bermejo JL (2004). Genetic epidemiology of cancer: from families to heritable genes International journal of cancer. J Int Cancer.

[CR19] Hoggart CJ, Whittaker JC, De Iorio M, Balding DJ (2008). Simultaneous analysis of all SNPs in genome-wide and re-sequencing association studies. PLoS Genet.

[CR20] Hoivik EA, Kusonmano K, Halle MK, Berg A, Wik E, Werner HM, Petersen K, Oyan AM, Kalland KH, Krakstad C (2014). Hypomethylation of the CTCFL/BORIS promoter and aberrant expression during endometrial cancer progression suggests a role as an Epi-driver gene. Oncotarget.

[CR21] Howie BN, Donnelly P, Marchini J (2009). A flexible and accurate genotype imputation method for the next generation of genome-wide association studies. PLoS Genet.

[CR22] James MA, Vikis HG, Tate E, Rymaszewski AL, You M (2014). CRR9/CLPTM1L regulates cell survival signaling and is required for Ras transformation and lung tumorigenesis. Cancer Res.

[CR23] Jia J, Bosley AD, Thompson A, Hoskins JW, Cheuk A, Collins I, Parikh H, Xiao Z, Ylaya K, Dzyadyk M (2014). CLPTM1L promotes growth and enhances aneuploidy in pancreatic cancer cells. Cancer Res.

[CR24] Jones AM, Howarth KM, Martin L, Gorman M, Mihai R, Moss L, Auton A, Lemon C, Mehanna H, Mohan H (2012). Thyroid cancer susceptibility polymorphisms: confirmation of loci on chromosomes 9q22 and 14q13, validation of a recessive 8q24 locus and failure to replicate a locus on 5q24. J Med Genet.

[CR25] Kaaks R, Lukanova A, Kurzer MS (2002). Obesity, endogenous hormones, and endometrial cancer risk: a synthetic review. Cancer Epidemiol Biomarkers Prev.

[CR26] Kandoth C, Schultz N, Cherniack AD, Akbani R, Liu Y, Shen H, Robertson AG, Pashtan I, Shen R, Benz CC (2013). Integrated genomic characterization of endometrial carcinoma. Nature.

[CR27] Kolquist KA, Ellisen LW, Counter CM, Meyerson M, Tan LK, Weinberg RA, Haber DA, Gerald WL (1998). Expression of TERT in early premalignant lesions and a subset of cells in normal tissues. Nat Genet.

[CR28] Kote-Jarai Z, Olama AA, Giles GG, Severi G, Schleutker J, Weischer M, Campa D, Riboli E, Key T, Gronberg H (2011). Seven prostate cancer susceptibility loci identified by a multi-stage genome-wide association study. Nat Genet.

[CR29] Kote-Jarai Z, Saunders EJ, Leongamornlert DA, Tymrakiewicz M, Dadaev T, Jugurnauth-Little S, Ross-Adams H, Al Olama AA, Benlloch S, Halim S (2013). Fine-mapping identifies multiple prostate cancer risk loci at 5p15, one of which associates with TERT expression. Hum Mol Genet.

[CR30] Landi MT, Chatterjee N, Yu K, Goldin LR, Goldstein AM, Rotunno M, Mirabello L, Jacobs K, Wheeler W, Yeager M (2009). A genome-wide association study of lung cancer identifies a region of chromosome 5p15 associated with risk for adenocarcinoma. Am J Hum Genet.

[CR31] Marchini J, Howie B (2010). Genotype imputation for genome-wide association studies. Nat Rev Genet.

[CR32] McKay JD, Hung RJ, Gaborieau V, Boffetta P, Chabrier A, Byrnes G, Zaridze D, Mukeria A, Szeszenia-Dabrowska N, Lissowska J (2008). Lung cancer susceptibility locus at 5p15.33. Nat Genet.

[CR33] Mhawech-Fauceglia P, Wang D, Kesterson J, Clark K, Monhollen L, Odunsi K, Lele S, Liu S (2010). Microarray analysis reveals distinct gene expression profiles among different tumor histology, stage and disease outcomes in endometrial adenocarcinoma. PLoS One.

[CR34] Michailidou K, Hall P, Gonzalez-Neira A, Ghoussaini M, Dennis J, Milne RL, Schmidt MK, Chang-Claude J, Bojesen SE, Bolla MK et al (2013) Large-scale genotyping identifies 41 new loci associated with breast cancer risk. Nat Genet 45:353–361, 361e351–352. doi:10.1038/ng.2563 10.1038/ng.2563 PMC3771688 23535729

[CR35] Moreno-Bueno G, Sanchez-Estevez C, Cassia R, Rodriguez-Perales S, Diaz-Uriarte R, Dominguez O, Hardisson D, Andujar M, Prat J, Matias-Guiu X (2003). Differential gene expression profile in endometrioid and nonendometrioid endometrial carcinoma: STK15 is frequently overexpressed and amplified in nonendometrioid carcinomas. Cancer Res.

[CR101] Painter JN, O'Mara TA, Batra J, Cheng T, Lose FA, Dennis J, Michailidou K, Tyrer JP, Ahmed S, Ferguson K et al (2014) Fine-mapping of the HNF1B multicancer locus identifies candidate variants that mediate endometrial cancer risk. Hum Mol Genet pii: ddu552 [Epub ahead of print] 10.1093/hmg/ddu552 PMC4321445 25378557

[CR36] Palles C, Cazier JB, Howarth KM, Domingo E, Jones AM, Broderick P, Kemp Z, Spain SL, Guarino E, Salguero I (2013). Germline mutations affecting the proofreading domains of POLE and POLD1 predispose to colorectal adenomas and carcinomas. Nat Genet.

[CR37] Petersen GM, Amundadottir L, Fuchs CS, Kraft P, Stolzenberg-Solomon RZ, Jacobs KB, Arslan AA, Bueno-de-Mesquita HB, Gallinger S, Gross M (2010). A genome-wide association study identifies pancreatic cancer susceptibility loci on chromosomes 13q22.1, 1q32.1 and 5p15.33. Nat Genet.

[CR38] Pharoah PD, Tsai YY, Ramus SJ, Phelan CM, Goode EL, Lawrenson K, Buckley M, Fridley BL, Tyrer JP, Shen H et al (2013) GWAS meta-analysis and replication identifies three new susceptibility loci for ovarian cancer. Nat Genet 45:362–370, 370e361–362. doi:10.1038/ng.2564 10.1038/ng.2564 PMC3693183 23535730

[CR39] Prescott J, McGrath M, Lee IM, Buring JE, De Vivo I (2010). Telomere length and genetic analyses in population-based studies of endometrial cancer risk. Cancer.

[CR40] Rafnar T, Sulem P, Stacey SN, Geller F, Gudmundsson J, Sigurdsson A, Jakobsdottir M, Helgadottir H, Thorlacius S, Aben KK (2009). Sequence variants at the *TERT–CLPTM1L* locus associate with many cancer types. Nat Genet.

[CR41] Risinger JI, Maxwell GL, Chandramouli GV, Jazaeri A, Aprelikova O, Patterson T, Berchuck A, Barrett JC (2003). Microarray analysis reveals distinct gene expression profiles among different histologic types of endometrial cancer. Cancer Res.

[CR42] Saidi SA, Holland CM, Kreil DP, MacKay DJ, Charnock-Jones DS, Print CG, Smith SK (2004). Independent component analysis of microarray data in the study of endometrial cancer. Oncogene.

[CR43] Sakoda LC, Jorgenson E, Witte JS (2013). Turning of COGS moves forward findings for hormonally mediated cancers. Nat Genet.

[CR44] Salvesen HB, Carter SL, Mannelqvist M, Dutt A, Getz G, Stefansson IM, Raeder MB, Sos ML, Engelsen IB, Trovik J (2009). Integrated genomic profiling of endometrial carcinoma associates aggressive tumors with indicators of PI3 kinase activation. Proc Natl Acad Sci USA.

[CR45] Setiawan VW, Doherty JA, Shu XO, Akbari MR, Chen C, De Vivo I, Demichele A, Garcia-Closas M, Goodman MT, Haiman CA (2009). Two estrogen-related variants in CYP19A1 and endometrial cancer risk: a pooled analysis in the Epidemiology of Endometrial Cancer Consortium. Cancer Epidemiol Biomarkers Prev.

[CR100] Setiawan VW, Haessler J, Schumacher F, Cote ML, Deelman E, Fesinmeyer MD, Henderson BE, Jackson RD, Vöckler JS, Wilkens LR (2012). HNF1B and endometrial cancer risk: results from the PAGE study. PLoS One.

[CR46] Shen H, Fridley BL, Song H, Lawrenson K, Cunningham JM, Ramus SJ, Cicek MS, Tyrer J, Stram D, Larson MC (2013). Epigenetic analysis leads to identification of HNF1B as a subtype-specific susceptibility gene for ovarian cancer. Nat Commun.

[CR47] Shen J, Gammon MD, Wu HC, Terry MB, Wang Q, Bradshaw PT, Teitelbaum SL, Neugut AI, Santella RM (2010). Multiple genetic variants in telomere pathway genes and breast cancer risk. Cancer Epidemiol Biomarkers Prev.

[CR48] Shete S, Hosking FJ, Robertson LB, Dobbins SE, Sanson M, Malmer B, Simon M, Marie Y, Boisselier B, Delattre JY (2009). Genome-wide association study identifies five susceptibility loci for glioma. Nat Genet.

[CR49] Spurdle AB, Thompson DJ, Ahmed S, Ferguson K, Healey CS, O’Mara T, Walker LC, Montgomery SB, Dermitzakis ET, Australian National Endometrial Cancer Study G (2011). Genome-wide association study identifies a common variant associated with risk of endometrial cancer. Nat Genet.

[CR50] Stacey SN, Sulem P, Masson G, Gudjonsson SA, Thorleifsson G, Jakobsdottir M, Sigurdsson A, Gudbjartsson DF, Sigurgeirsson B, Benediktsdottir KR (2009). New common variants affecting susceptibility to basal cell carcinoma. Nat Genet.

[CR51] Supernat A, Lapinska-Szumczyk S, Sawicki S, Wydra D, Biernat W, Zaczek AJ (2012). Deregulation of RAD21 and RUNX1 expression in endometrial cancer. Oncol Lett.

[CR52] Thomas G, Jacobs KB, Yeager M, Kraft P, Wacholder S, Orr N, Yu K, Chatterjee N, Welch R, Hutchinson A (2008). Multiple loci identified in a genome-wide association study of prostate cancer. Nat Genet.

[CR53] Turnbull C, Rapley EA, Seal S, Pernet D, Renwick A, Hughes D, Ricketts M, Linger R, Nsengimana J, Deloukas P (2010). Variants near DMRT1, TERT and ATF7IP are associated with testicular germ cell cancer. Nat Genet.

[CR54] Tyrer J, Pharoah PD, Easton DF (2006). The admixture maximum likelihood test: a novel experiment-wise test of association between disease and multiple SNPs. Genet Epidemiol.

[CR55] Vignal CM, Bansal AT, Balding DJ (2011). Using penalised logistic regression to fine map HLA variants for rheumatoid arthritis. Ann Hum Genet.

[CR56] Wang Y, Broderick P, Webb E, Wu X, Vijayakrishnan J, Matakidou A, Qureshi M, Dong Q, Gu X, Chen WV (2008). Common 5p15.33 and 6p21.33 variants influence lung cancer risk. Nat Genet.

[CR57] Wang Z, Zhu B, Zhang M, Parikh H, Jia J, Chung CC, Sampson JN, Hoskins JW, Hutchinson A, Burdette L et al (2014) Imputation and subset-based association analysis across different cancer types identifies multiple independent risk loci in the *TERT–CLPTM1L* region on chromosome 5p15.33. Hum Mol Genet. doi:10.1093/hmg/ddu363 10.1093/hmg/ddu363 PMC4240198 25027329

[CR58] Ward LD, Kellis M (2012). HaploReg: a resource for exploring chromatin states, conservation, and regulatory motif alterations within sets of genetically linked variants. Nucleic Acids Res.

[CR59] Wellcome Trust Case Control C (2007). Genome-wide association study of 14,000 cases of seven common diseases and 3,000 shared controls. Nature.

[CR60] Yamamoto K, Okamoto A, Isonishi S, Ochiai K, Ohtake Y (2001). A novel gene, CRR9, which was up-regulated in CDDP-resistant ovarian tumor cell line, was associated with apoptosis. Biochem Biophys Res Commun.

[CR61] Zighelboim I, Mutch DG, Knapp A, Ding L, Xie M, Cohn DE, Goodfellow PJ (2014). High frequency strand slippage mutations in CTCF in MSI-positive endometrial cancers. Hum Mutat.

