# Supplementary material for: Candidate locus analysis of the TERT–CLPTM1L cancer risk region on chromosome 5p15 identifies multiple independent variants associated with endometrial cancer risk
Source: Hum Genet. 2014 Dec 9;134(2):231–45. doi: 10.1007/s00439-014-1515-4 (PMC4291520; doi:10.1007/s00439-014-1515-4)
Supplement: Supplementary file 1 — Supplementary material 1 (PDF 259 kb) [file 439_2014_1515_MOESM1_ESM.pdf]

Multiple independent variants at and around the *TERT-CLPTM1L* candidate region on chromosome 5p15 are associated with endometrial cancer risk. Human Genetics. Luis Carvajal-Carmona et al. Email: lgcarvajal@ucdavis.edu

Supplementary Figure 1.

A. rs7705526

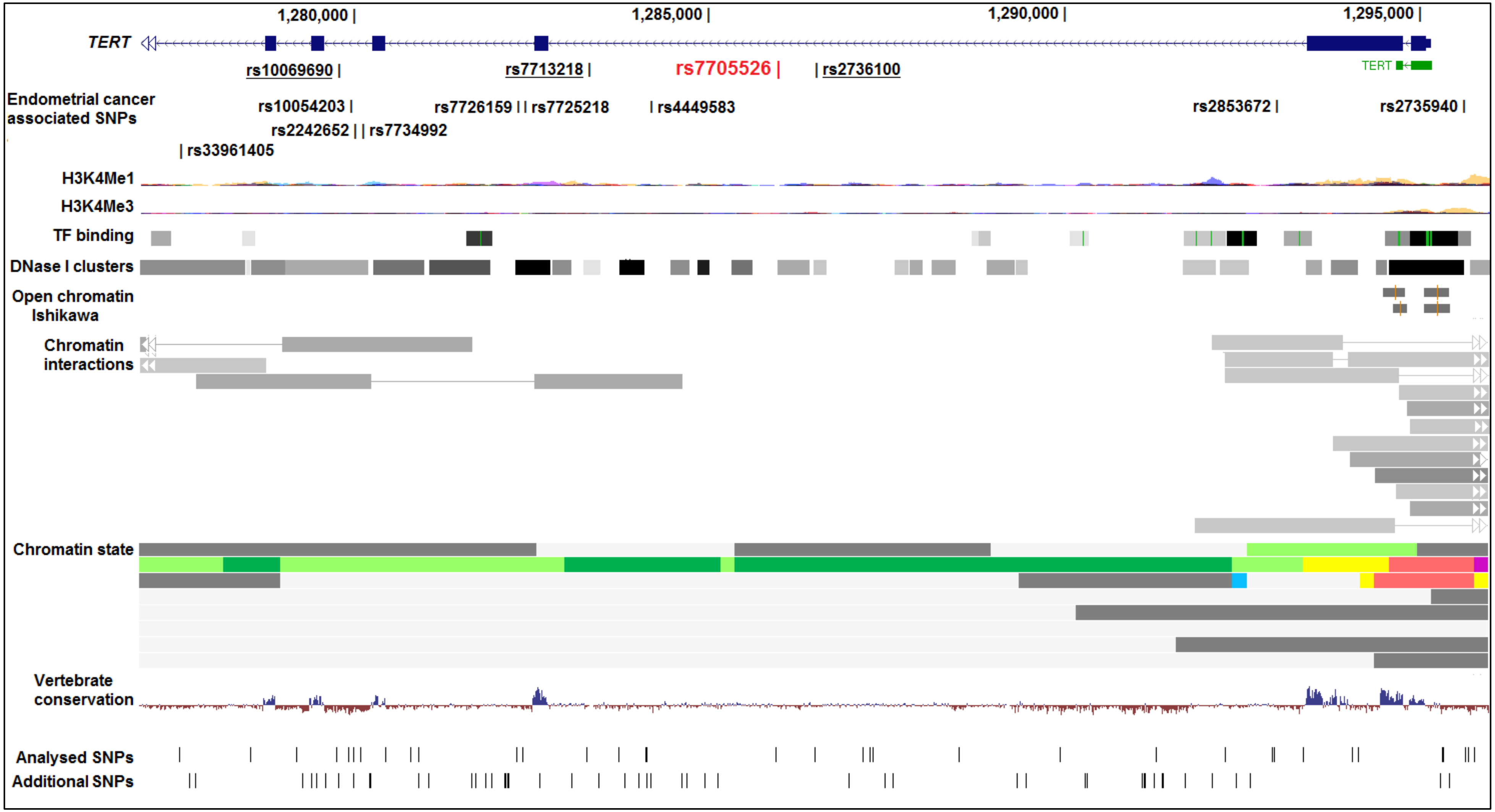

B. rs13174814

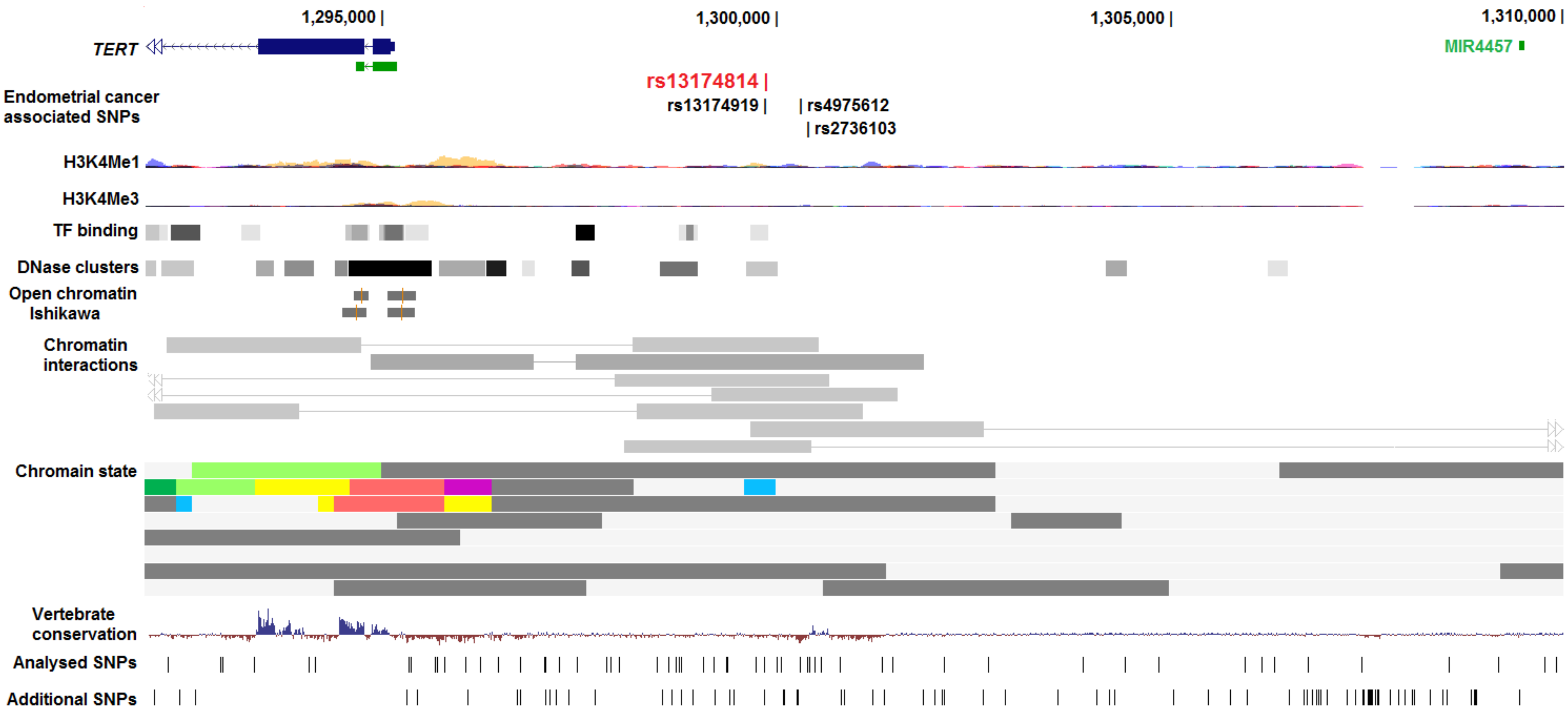

C. rs62329728

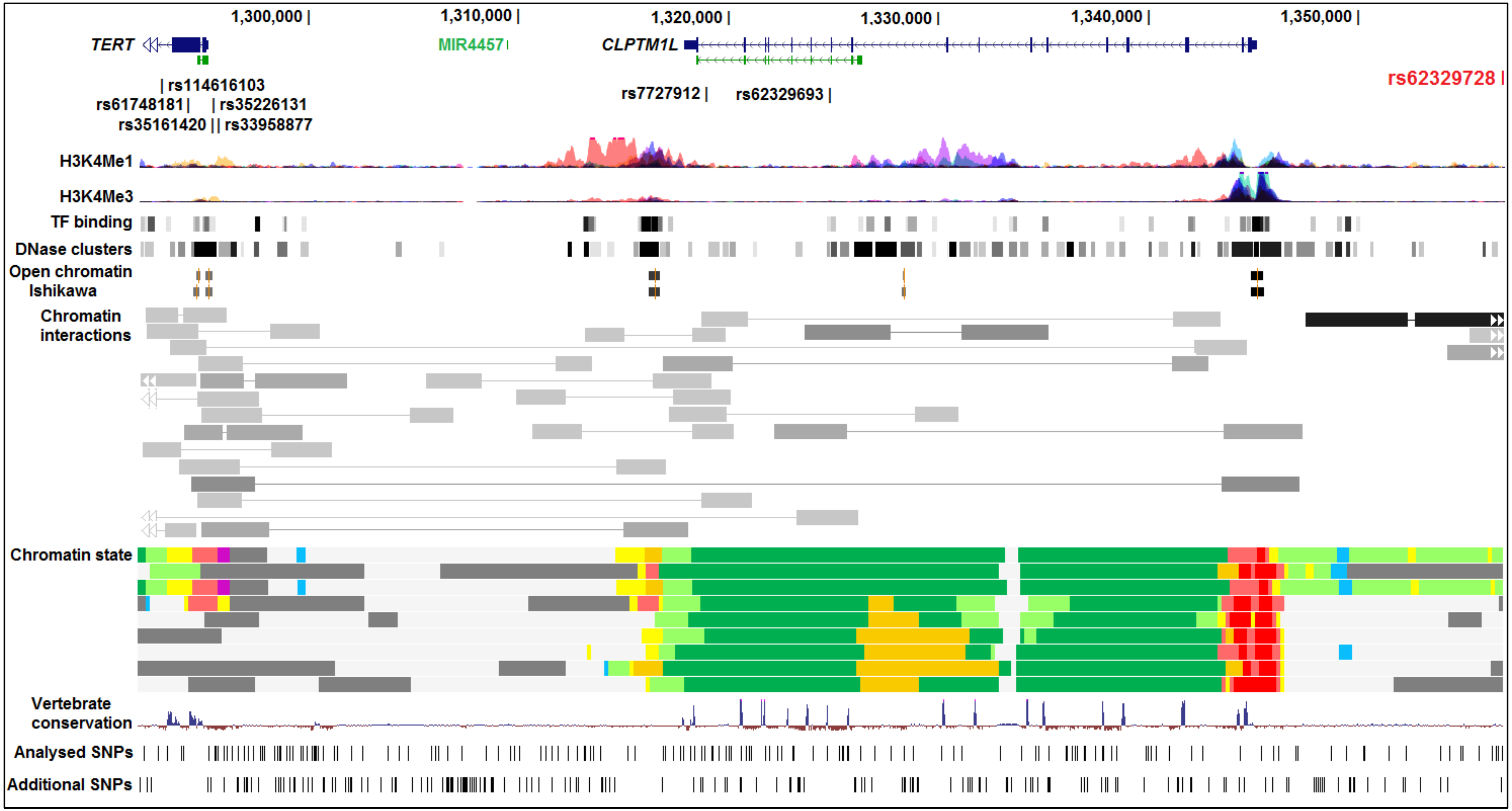

Supplementary Figure 1. Epigenetic landscape at the *TERT-CLPTM1L* locus for association peaks centered on the endometrial cancer lead SNPs **A.** rs7705526; **B.** rs13174814; **C.** rs62329728. For each panel, the location of lead SNPs in relation to the closest gene (*TERT* or *CLPTM1L*) are indicated in red and additional SNPs in high linkage disequilibrium ($r^2 \geq 0.8$) with each lead SNP are indicated in black. SNPs known to be associated with other cancer types are underlined (Panel A. only). The most likely causal SNPs in each peak are shown in relation to marks of regulatory potential as indicated by data obtained through ENCODE: Histones **H3K4Me1** (indicative of regulatory regions) and **H3K4Me3** (indicative of promoters); **TF binding** (transcription factor binding) in 72 ENCODE cell lines; **DNase clusters** defined by *DNaseI* hypersensitivity (indicative of open chromatin, with darker shading indicating stronger experimental signal) in 125 ENCODE cell lines; **Open chromatin in Ishikawaka** endometrial cancer cell lines (treated with 4-OHTAM and estradiol 10m); **Chromatin interactions** in K562 and MCF7 cancer cell lines; **Chromatin state** in multiple ENCODE cell lines, where bright red-active promoter; light red-weak promoter; purple-inactive/poised promoter; orange-strong enhancer; yellow-weak enhancer; blue-insulator; dark green-transcriptional transition; light green-weak transcribed; dark gray-repressed/heterochromatin (http://genome.ucsc.edu/ENCODE/); **Vertebrate conservation** across 100 species; **Analysed SNPs** included in the current analysis; **Additional SNPs** in the region (included to show total SNP coverage in the region and the presence/absence of substantial gaps in coverage).
