# Supplementary figures and images for: Candidate locus analysis of the TERT–CLPTM1L cancer risk region on chromosome 5p15 identifies multiple independent variants associated with endometrial cancer risk
Source: Hum Genet. 2014 Dec 9;134(2):231–45. doi: 10.1007/s00439-014-1515-4 (PMC4291520; doi:10.1007/s00439-014-1515-4)

**UK GWAS info**

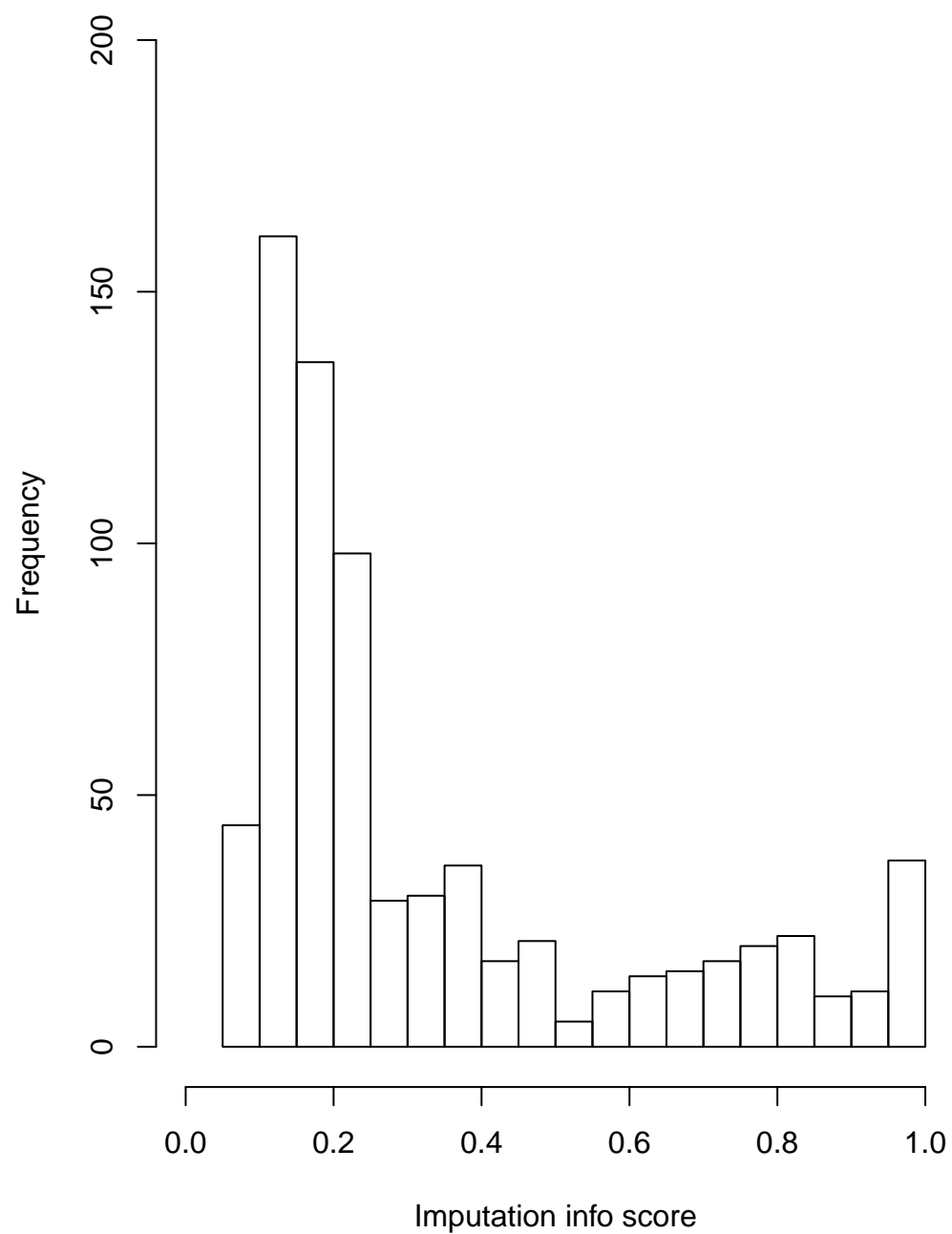

**iCOGS info**

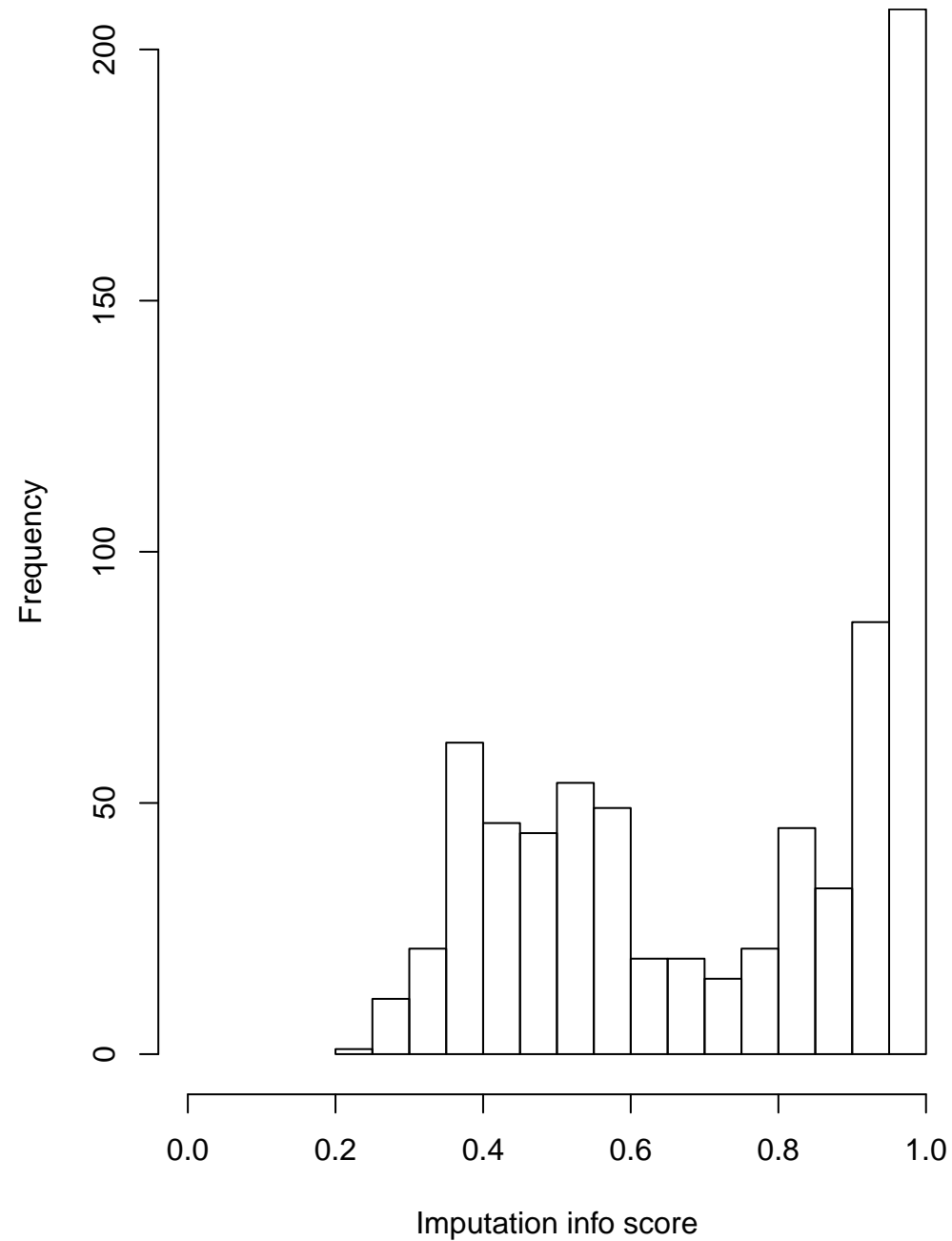

Supplement: Supplementary file 2 — Supplementary Fig. 2: Histograms comparing the SNP coverage of the chromosome 5 1,200,000–1,400,000 region by the Illuminia Infinium 1.2 M GWAS array (as genotyped in 5180 control subjects from the Wellcome Trust Case Control Consortium) with that of the Illuminia iSelect iCOGS array genotyped in this study, as captured by the quality of imputation to the 1000 Genomes April 2012 reference panel (PDF 4 kb) [file 439_2014_1515_MOESM2_ESM.pdf]
