# Supplementary material for: Candidate locus analysis of the TERT–CLPTM1L cancer risk region on chromosome 5p15 identifies multiple independent variants associated with endometrial cancer risk
Source: Hum Genet. 2014 Dec 9;134(2):231–45. doi: 10.1007/s00439-014-1515-4 (PMC4291520; doi:10.1007/s00439-014-1515-4)
Supplement: Supplementary file 4 — Supplementary material 4 (DOC 124 kb) [file 439_2014_1515_MOESM4_ESM.doc]

**Human Genetics: Supplementary Information for “Candidate locus analysis of the** ***TERT*-*CLPTM1L* cancer risk region on chromosome 5p15 identifies multiple independent variants associated with endometrial cancer risk**

Luis G Carvajal-Carmona^1^*, Tracy A O’Mara^2^, Jodie N Painter^2^, Felicity A Lose^2^, Joe Dennis^3^, Kyriaki Michailidou^3^, Jonathan P Tyrer^4^, Shahana Ahmed^4^, Kaltin Ferguson^2^, Catherine S Healey^4^, Karen Pooley^3^, Jonathan Beesley^2^, Timothy Cheng^5^, Angela Jones^5^, Kimberley Howarth^5^, Lynn Martin^5^, Maggie Gorman^5^, Shirley Hodgson^6^, National Study of Endometrial Cancer Genetics Group (NSECG)^5,7^, The Australian National Endometrial Cancer Study Group (ANECS)^2,7^, Nicholas Wentzensen^8^, Peter A Fasching^9,10^, Alexander Hein^10^, Matthias W Beckmann^10^, Stefan P Renner^10^, Thilo Dörk^11^, Peter Hillemanns^12^, Matthias Dürst^13^, Ingo Runnebaum^13^, Diether Lambrechts^14,15^, Lieve Coenegrachts^16^, Stefanie Schrauwen^16^, Frederic Amant^16,17^, Boris Winterhoff^18^, Sean C Dowdy^18^, Ellen L Goode^19^, Attila Teoman^18^, Helga B Salvesen^20,21^, Jone Trovik^20,21^, Tormund S Njolstad^20,21^, Henrica MJ Werner^20,21^, Rodney J Scott^22–24^, Katie Ashton^22,24,25^, Tony Proietto^26^, Geoffrey Otton^26^, Gerasimos Tzortzatos^27^, Miriam Mints^27^, Emma Tham^28^, RENDOCAS^7,28^, Per Hall^29^, Kamila Czene^29^, Jianjun Liu^30^, Jingmei Li^30^, John L Hopper^31^, Melissa C Southey^32^, Australian Ovarian Cancer Study (AOCS)^2,7,33^, Arif B Ekici^10^, Matthias Ruebner^10^, Nichola Johnson^34^, Julian Peto^35^, Barbara Burwinkel^36,37^, Frederik Marme^36,38^, Hermann Brenner^37,38^, Aida K Dieffenbach^37,38^, Alfons Meindl^39^, Hiltrud Brauch^40^, The GENICA Network^7,40-45^, Annika Lindblom^28^, Jeroen Depreeuw^14,15^, Matthieu Moisse^14,15^, Jenny Chang-Claude^46^, Anja Rudolph^47^, Fergus J Couch^48^, Janet E Olson^19^, Graham G Giles^31,49,50^, Fiona Bruinsma^49^, Julie M Cunningham^48^, Brooke L Fridley^51^, Anne-Lise Børresen-Dale^52,53^, Vessela N Kristensen^52-54^, Angela Cox^55^, Anthony J Swerdlow^56,57^, Nicholas Orr^57^, Manjeet K Bolla^3^, Qin Wang^3^, Rachel Palmieri Weber^58^, Zhihua Chen^59^, Mitul Shah^4^, Paul D P Pharoah^4^, Alison M Dunning^4^, Ian Tomlinson^5^¶, Douglas F Easton^3,4^¶, Amanda B Spurdle^2^¶, Deborah J Thompson^3^*

1. Genome Center and Department of Biochemistry and Molecular Medicine, School of Medicine, University of California, Davis, USA.

2. QIMR Berghofer Medical Research Institute, Brisbane Queensland, Australia.

3. Centre for Cancer Genetic Epidemiology, Department of Public Health and Primary Care, University of Cambridge, Cambridge, UK.

4. Centre for Cancer Genetic Epidemiology, Department of Oncology, University of Cambridge, Cambridge, UK.

5. Wellcome Trust Centre for Human Genetics, University of Oxford, Oxford, UK.

6. Department of Clinical Genetics, St George’s Hospital Medical School, London, UK.

7. See Supplementary Note for full list of members

8. Division of Cancer Epidemiology and Genetics, National Cancer Institute, USA.

9. University of California at Los Angeles, Department of Medicine, Division of Hematology/Oncology, David Geffen School of Medicine, Los Angeles, CA, UK.

10. University Hospital Erlangen, Department of Gynecology and Obstetrics, Friedrich-Alexander University Erlangen-Nuremberg, Erlangen, Germany.

11. Hannover Medical School, Gynaecology Research Unit, Hannover, Germany.

12. Hannover Medical School, Clinics of Gynaecology and Obstetrics, Hannover, Germany.

13. Department of Gynaecology, Jena University Hospital - Friedrich Schiller University, Jena, Germany.

14. Vesalius Research Center, VIB, Leuven, Belgium.

15. Laboratory for Translational Genetics, Department of Oncology, KU Leuven, Belgium.

16. Department of Oncology, KU Leuven, Belgium.

17. Division of Gynaecological Oncology, University Hospital Leuven, Leuven, Belgium.

18. Department of Obstetrics and Gynecology, Division of Gynecologic Oncology, Mayo Clinic, Rochester, MN, USA.

19. Department of Health Science Research, Division of Epidemiology, Mayo Clinic, Rochester, MN, USA.

20. Centre for Cancerbiomarkers, Department of Clinical Science, The University of Bergen, Norway.

21. Department of Obstetrics and Gynecology, Haukeland University Hospital, Bergen, Norway.

22. Hunter Medical Research Institute, John Hunter Hospital, Newcastle, NSW, Australia.

23. Hunter Area Pathology Service, John Hunter Hospital, Newcastle, NSW, Australia.

24. Centre for Information Based Medicine, School of Biomedical Science and Pharmacy, University of Newcastle, NSW, Australia.

25. Discipline of Medical Genetics, School of Biomedical Sciences and Pharmacy, University of Newcastle, NSW, Australia.

26. School of Medicine and Public Health, University of Newcastle, NSW, Australia.

27. Department of Women’s and Children's Health, Karolinska Institutet, Karolinska University Hospital, Stockholm, Sweden.

28. Department of Molecular Medicine and Surgery, Karolinska Institutet, Stockholm, Sweden.

29. Department of Medical Epidemiology and Biostatistics, Karolinska Institutet, Stockholm, Sweden.

30. Human Genetics, Genome Institute of Singapore, Singapore.

31. Centre for Epidemiology and Biostatistics, Melbourne School of Population and Global Health, The University of Melbourne, Victoria, Australia.

32. Genetic Epidemiology Laboratory, Department of Pathology, The University of Melbourne, Melbourne, Victoria, Australia.

33. Peter MacCullum Cancer Centre, Melbourne, Victoria, Australia.

34. Breakthrough Breast Cancer Research Centre, Institute of Cancer Research, London, UK.

35. London School of Hygiene and Tropical Medicine, London, UK.

36. Molecular Biology of Breast Cancer, Department of Gynecology and Obstetrics, University of Heidelberg, Heidelberg, Germany.

37. Division of Clinical Epidemiology and Aging Research, German Cancer Research Center (DKFZ), Heidelberg, Germany.

38. German Cancer Consortium (DKTK), Heidelberg, Germany.

39. Department of Obstetrics and Gynecology, Division of Tumor Genetics, Technical University of Munich, Munich, Germany.

40. Dr. Margarete Fischer-Bosch Institute of Clinical Pharmacology Stuttgart, University of Tuebingen, Germany.

41. Institute for Occupational Medicine and Maritime Medicine, University Medical Center Hamburg-Eppendorf, Germany.

42. Department of Internal Medicine, Evangelische Kliniken Bonn gGmbH, Johanniter Krankenhaus, Bonn, Germany.

43. Institute of Pathology, Medical Faculty of the University of Bonn, Bonn, Germany.

44. Institute for Prevention and Occupational Medicine of the German Social Accident Insurance, Institute of the Ruhr University Bochum (IPA), Bochum, Germany.

45. Molecular Genetics of Breast Cancer, Deutsches Krebsforschungszentrum (DKFZ), Heidelberg, Germany.

46. Division of Cancer Epidemiology, German Cancer Research Center, Heidelberg, Germany.

47. Department of Cancer Epidemiology/Clinical Cancer Registry and Institute for Medical Biometrics and Epidemiology, University Clinic Hamburg-Eppendorf, Hamburg, Germany.

48. Departments of Laboratory Medicine and Pathology, and Health Science Research, Mayo Clinic, Rochester, MN, USA.

49. Cancer Epidemiology Centre, Cancer Council Victoria, Melbourne, Australia.

50. Department of Epidemiology and Preventive Medicine, Monash University, Melbourne, Australia.

51. Department of Biostatistics, University of Kansas Medical Center, Kansas City, KS, USA.

52. Department of Genetics, Institute for Cancer Research, The Norwegian Radium Hospital, Oslo, Norway.

53. The K.G. Jebsen Center for Breast Cancer Research, Institute for Clinical Medicine, Faculty of Medicine, University of Oslo, Oslo, Norway.

54. Department of Clinical Molecular Oncology, Division of Medicine, Akershus University Hospital, Ahus, Norway.

55. Sheffield Cancer Research Centre, Department of Oncology, University of Sheffield, UK.

56. Division of Genetics and Epidemiology, Institute of Cancer Research, London, UK.

57. Division of Breast Cancer Research, Institute of Cancer Research, London, UK.

58. Department of Community and Family Medicine, Duke University School of Medicine, Durham, NC, USA.

59. Department of Cancer Epidemiology, Division of Population Sciences, Moffitt Cancer Center, Tampa, Florida, USA.

^¶^ These authors contributed equally to the study

* To whom correspondence should be addressed:

Genome Center, University of California, Davis, California 95616, USA. Tel: +1 5307529654, Fax: +1 530 754 9658, Email: [lgcarvajal@ucdavis.edu](mailto:lgcarvajal@ucdavis.edu) (LGC-C) or

Centre for Cancer Genetic Epidemiology, University of Cambridge, Strangeways Research Laboratory, Worts Causeway, Cambridge, CB1 8RN, United Kingdom. Tel: +44 (0)1223 748631, Fax: +44 (0)1223 748628, Email: [djt25@medschl.cam.ac.uk](mailto:djt25@medschl.cam.ac.uk) (DJT)

**INDEX**

A. Detailed Description of the Case and Control Sample Sets 5

B

. ECAC Study Collaborators 7

C. BCAC and OCAC Study Collaborators (for control samples): 8

D. Supplementary Acknowledgements 8

E. Additional Acknowledgments of Funding to BCAC/OCAC control groups 10

**SUPPLEMENTARY NOTE**

## A. Detailed Description of the Case and Control Sample Sets

A summary of the studies included in the iCOGS fine-mapping dataset is shown in **Supplementary Table 1**, with additional details provided below. All studies were predominantly of women of European ancestry. All studies have the relevant IRB approval in each country in accordance with the principles embodied in the Declaration of Helsinki, and informed consent was obtained from all participants.

***Fine-mapping (iCOGS) Case Sample Sets:***

The iCOGS fine-mapping data set included cases from 9 studies detailed below, as well as additional European ancestry cases from ANECS and SEARCH (non-overlapping with the GWAS datasets).

**ANECS**

The Australian National Endometrial Cancer Study (ANECS) is an Australian population-based case-control family study of cancer of the uterine corpus. Women aged 18-79, newly diagnosed with histologically confirmed primary cancer of the endometrium between July 2005 and December 2007 were identified through major hospitals nationally, and also from state-based cancer registries. Excluding women who could not be contacted (mostly due to death, illness or failure to contact), case participation rate was 63%. Participants completed a detailed questionnaire providing clinical and epidemiological information, including ethnicity of all four grandparents. Information on tumor pathology characteristics was abstracted in standardized format from clinical pathology reports for all patients. Samples included in the iCOGS fine-mapping genotyping analysis did not overlap those included in stage 1 of a previously published endometrioid endometrial cancer GWAS .

**BECS**

The Bavarian Endometrial Cancer Cases and Controls Study (BECS) is a single-center case-control study, conducted between 2002 and 2008, with the aim of investigating genetic and epidemiological risk factors for endometrial cancer. Cases were either incident cases referred to the University Hospital Erlangen by surrounding practioners (66% of the case sample set), or prevalent cases that were outpatients in follow-up care approached within 6.2 (±4.6 SD) years after treatment for primary endometrial cancer in the same hospital (34% of the case sample set). Epidemiological information was collected by a structured questionnaire completed during an interview and clinical data for the cases was obtained from clinical health records.

**CAHRES**

Details of the population selection process have been published previously for the Cancer Hormone Replacement Epidemiology Study (CAHRES). Formerly known as the Singapore and Sweden Breast/Endometrial Cancer Study (SASBAC), this population based case-control study was conducted among Swedish women aged 50-74 years, who were residing in Sweden between January 1^st^ 1994 and December 31^st^ 1995. Endometrial cancer cases were identified through the nation-wide cancer registries in Sweden. All participants provided detailed questionnaire information. For endometrial cancer, histological specimens were reviewed and re-classified by the study pathologist. All participants reported Caucasian ethnicity.

**HJECS**

The Hannover-Jena Endometrial Cancer Study (HJECS), a hospital-based case-control study, included 250 German women, aged 31-89 years, who were recruited either at the Friedrich Schiller University of Jena or at Hannover Medical School after having been diagnosed with histologically confirmed primary incident endometrial carcinoma between 2004 and 2010. Epidemiological data were obtained from questionnaires, and information on tumor stage and histology was obtained from pathology and clinical reports. Over 98% were of German descent. Interviews were conducted at either the Friedrich Schiller University of Jena or at Hannover Medical School, and peripheral blood was collected for the extraction of DNA from white blood cells.

**LES**

The Leuven Endometrial Study (LES) is a hospital based case-control study. Eligible cases, identified by active surveillance of electronic patient files at the Leuven University Hospital, were white women aged 27-80 years diagnosed with endometrial cancer. Clinical data for endometrial cancer patients were recorded during interview at the time of diagnosis, and from pathology reports. All medical records were reviewed by trained abstractors and pathology reports compatible with primary, invasive, epithelial endometrial adenocarcinoma of all stages (I –IV) and all grades were consulted. Participation rates exceeded 95% for cases.

**MECS**

The Mayo Endometrial Cancer Study (MECS) is a hospital based prospective biobank collection. The majority of patients seen at Mayo Clinic Rochester with primary endometrial cancer diagnosed at age 18 and older are enrolled. The collection was started in 2006 and contains blood and fresh frozen tissue. DNA was isolated from white blood cells using Qiagen isolation kits. DNA concentration was measured with picogreen. Clinical data were abstracted from electronic medical records. Control data were obtained from Mayo Clinic OCAC controls.

**MoMaTEC**

Molecular Markers in Treatment of Endometrial Cancer (MoMaTEC) cases were recruited from an unselected patient population primarily treated for endometrial carcinoma at Haukeland University Hospital, Bergen during 2001-2009. This is the referral hospital for Hordaland county; the area is demographically well defined, with about 450,000 inhabitants, representing approximately 10% of the Norwegian population and with a similar incidence rate and prognosis as the total Norwegian population of endometrial cancers. Clinical Information for cases regarding age, FIGO stage, histologic subtype, grade and prognosis was extracted from medical records.DNA was extracted from peripheral blood samples.

**NECS**

The Newcastle Endometrial Cancer Study (NECS) includes histologically confirmed endometrial cancer cases consecutively recruited from 1992 up to 2005 at the Hunter Centre for Gynaecological Cancer, John Hunter Hospital, Newcastle, New South Wales, Australia. The final analysis included 194 endometrial cancer patients. Data on reproductive and environmental risk factors including ethnicity, was collected using self-reported questionnaires. Information regarding recurrence, stage, grade and histology of endometrial cancer was collected from medical records. Patients presenting at this hospital-based site were captured by ANECS recruitment from 2005 onwards.

**NSECG**

National Study of the Genetics of Endometrial Cancer (NSECG) cases were identified from collaborating clinicians throughout the UK from 2008 to present, taking care not to recruit from centres involved in SEARCH. Inclusion criteria were adenocarcinomas of the uterus presenting at 70 years of age or younger. Almost all cases were incident and sampled within 6 months of diagnosis. Peripheral blood was collected from each participant and DNA extracted using standard methods. Tumor histology was confirmed from routine hospital reports and further details of histopathology and other tumor pathology characteristic was abstracted from these clinical pathology reports.

**RENDOCAS**

The Registry of Endometrial Cancer in Sweden (RENDOCAS) is a hospital based case-control study. Patients (n=262) who underwent surgery for endometrial cancer at Karolinska University hospital Solna, Sweden between 2008 and 2011 were included in the study. For each patient, the following was collected: blood and tumor samples; detailed family history and formulation of a pedigree where all suspected cancer cases were verified in medical records/pathology report if possible; questionnaire covering relevant environmental factors underlying endometrial cancer.

**SEARCH**

The Studies of Epidemiology and Risk factors in Cancer Heredity (SEARCH) is an ongoing population-based study with cases ascertained through the Eastern Cancer Registration and Information Centre ([http://www.ecric.org.uk](http://www.ecric.org.uk/)). All women diagnosed with endometrial cancer between the ages of 18-69 years (average age of diagnosis 58 years) from August 2001 to September 2007 were eligible for inclusion. Approximately 54% of eligible patients have enrolled in the study. Women taking part in the study were asked to provide a 20ml blood sample for DNA analysis, and to complete a comprehensive epidemiological questionnaire. Controls were also drawn from SEARCH (http://ccge.medschl.cam.ac.uk/search/), but had no prior history of cancer at the time of recruitment. They were female, also between the ages of 18-69 at the time of recruitment and matched to cases in geographical profile. Approximately 35% of eligible controls enrolled in the study. All participants reported Caucasian ethnicity. Information on tumor pathology characteristics was provided by the Eastern Cancer Registration and Information Centre and was derived from clinical pathology reports for all patients. Samples included in the iCOGS fine-mapping genotyping analysis did not overlap those included in stage 1 of a previously published endometrioid endometrial cancer GWAS .

**Control sample sets**

As indicated in **Supplementary Table 1**, endometrial cancer case sample sets were matched by country to combined control sample sets from the same country that had been genotyped using the iCOGS chip. Data was largely from control sample sets that participated in the Breast Cancer Association Consortium iCOGS experiment, with iCOGS data also accessed for controls from the Mayo Clinic via the Ovarian Cancer Association Consortium (MAY). In addition, iCOGS genotyping was performed for 183 Norwegian female controls, recruited in Bergen via the blood bank specifically for use in the MoMaTEC case-control genotyping studies.

## B. ECAC Study Collaborators

***The ANECS Group comprises****:* AB Spurdle, PM Webb, J Young (QIMR Berghofer Medical Research Institute); Consumer representative: L McQuire; Clinical Collaborators: NSW: S Baron-Hay, D Bell, A Bonaventura, A Brand, S Braye, J Carter, F Chan, C Dalrymple, A Ferrier (deceased), G Gard, N Hacker, R Hogg, R Houghton, D Marsden, K McIlroy, G Otton, S Pather, A Proietto, G Robertson, J Scurry, R Sharma, G Wain, F Wong; Qld: J Armes, A Crandon, M Cummings, R Land, J Nicklin, L Perrin, A Obermair, B Ward; SA: M Davy, T Dodd, J Miller, M Oehler, S Paramasivum, J Pierides, F Whitehead; Tas: P Blomfield, D Challis; Vic: D Neesham, J Pyman, M Quinn, R Rome, M Weitzer; WA: B Brennan, I Hammond, Y Leung, A McCartney (deceased), C Stewart, J Thompson; Project Managers: S O'Brien, S Moore; Laboratory Manager: K Ferguson; Pathology Support: M Walsh; Admin Support: R Cicero, L Green, J Griffith, L Jackman, B Ranieri; Laboratory Assistants: M O'Brien, P Schultz; Research Nurses: B Alexander, C Baxter, H Croy, A Fitzgerald, E Herron, C Hill, M Jones, J Maidens, A Marshall, K Martin, J Mayhew, E Minehan, D Roffe, H Shirley, H Steane, A Stenlake, A Ward, S Webb, J White.

***NECS collaborators include:*** Ute Hamann and Michael Gilbert.

***The NSECG Group comprises:*** Ian Tomlinson (Oxford University); M Adams, A Al-Samarraie, S Anwar, R Athavale, S Awad, A Bali, A Barnes, G Cawdell, S Chan, K Chin, P Cornes, M Crawford, J Cullimore, S Ghaem-Maghami, R Gornall, J Green, M Hall, M Harvey, J Hawe, A Head, J Herod, M Hingorani, M Hocking, C Holland, T Hollingsworth,J Hollingworth, T Ind, R Irvine, C Irwin, M Katesmark, S Kehoe, G Kheng-Chew, K Lankester, A Linder, D Luesley, C B-Lynch,V McFarlane, R Naik, N Nicholas, D Nugent, S Oates, A Oladipo, A Papadopoulos, S Pearson, D Radstone, S Raju, A Rathmell, C Redman, M Rymer, P Sarhanis, G Sparrow, N Stuart, S Sundar, A Thompson, S Tinkler, S Trent, A Tristram, N Walji, R Woolas.

***RENDOCAS investigators include***: Annika Lindblom, Gerasimos Tzortzatos, Miriam Mints, Emma Tham, Ofra Castro, Kristina Gemzell-Danielsson.

***SEARCH collaborators include:*** Helen Baker, Caroline Baynes, Don Conroy, Bridget Curzon, Patricia Harrington, Sue Irvine, Craig Luccarini, Rebecca Mayes, Hannah Munday, Barbara Perkins, Daisy Pharoah, Radka Platte, Anne Stafford and Judy West.

## C. BCAC and OCAC Study Collaborators (for control samples):

***The Australian Ovarian Cancer Study Group comprises:*** R Stuart-Harris; NSW‐ F Kirsten, J Rutovitz, P Clingan, A Glasgow, A Proietto, S Braye, G Otton, J Shannon, T Bonaventura, J Stewart, S Begbie, M Friedlander, D Bell, S Baron-­Hay, A Ferrier (deceased), G Gard, D Nevell, N Pavlakis, S Valmadre, B Young, C Camaris, R Crouch, L Edwards, N Hacker, D Marsden, G Robertson, P Beale, J Beith, J Carter, C Dalrymple, R Houghton, P Russell, L Anderson, M Links, J Grygiel, J Hill, A Brand, K Byth, R Jaworski, P Harnett, R Sharma, G Wain; QLD- D Purdie, D Whiteman, B Ward, D Papadimos, A Crandon, M Cummings, K Horwood. A Obermair, L Perrin, D Wyld, J Nicklin; SA- M Davy, MK Oehler, C Hall, T Dodd, T Healy, K Pittman, D Henderson, J Miller, J Pierdes, A Achan; TAS- P Blomfield, D Challis, R McIntosh, A Parker; VIC- B Brown, R Rome, D Allen, P Grant, S Hyde, R Laurie M Robbie, D Healy, T Jobling, T Manolitsas, J McNealage, P Rogers, B Susil, E Sumithran, I Simpson, I Haviv, K Phillips, D Rischin, S Fox, D Johnson, S Lade, P Waring, M Loughrey, N O’Callaghan, B Murray, L Mileshkin, P Allan; V Billson, J Pyman, D Neesham, M Quinn, A Hamilton, C Underhill, R Bell, LF Ng, R Blum, V Ganju; WA- I Hammond, A McCartney (deceased), C Stewart, Y Leung, M Buck, N Zeps (WARTN); AOCS Management Group- DDL Bowtell, AC Green, G Chenevix-Trench, A deFazio, D Gertig, PM Webb.

***BSUCH collaborator:*** Peter Bugert

***ESTHER collaborators:*** Volker Arndt, Heiko Müller, Christa Stegmaier

***GENICA Network collaborators***: Wing-Yee Lo, Christina Justenhoven, Ute Hamann, Thomas Brüning, Beate Pesch, Yon-Dschun Ko, Sylvia Rabstein, Anne Lotz, Christina Baisch, Hans-Peter Fischer, Volker Harth.

## D. Supplementary Acknowledgements

The authors thank the many individuals who participated in this study and the numerous institutions and their staff who have supported recruitment.

ANECS thanks members of the Molecular Cancer Epidemiology and Cancer Genetic laboratories at QIMR Berfhofer Medical Research Institute for technical assistance, and the ANECS research team for assistance with the collection of risk factor information and blood samples. ANECS also gratefully acknowledges the cooperation of the following institutions: NSW: John Hunter Hospital, Liverpool Hospital, Mater Misericordiae Hospital (Sydney), Mater Misericordiae Hospital (Newcastle), Newcastle Private Hospital, North Shore Private Hospital, Royal Hospital for Women, Royal Prince Alfred Hospital, Royal North Shore Hospital, Royal Prince Alfred Hospital, St George Hospital; Westmead Hospital, Westmead Private Hospital; Qld: Brisbane Private Hospital, Greenslopes Hospital, Mater Misericordiae Hospitals, Royal Brisbane and Women's Hospital, Wesley Hospital, Queensland Cancer Registry; SA: Adelaide Pathology Partners, Burnside Hospital, Calvary Hospital, Flinders Medical Centre, Queen Elizabeth Hospital, Royal Adelaide Hospital, South Australian Cancer Registry; Tas: Launceston Hospital, North West Regional Hospitals, Royal Hobart Hospital; Vic: Freemasons Hospital, Melbourne Pathology Services, Mercy Hospital for Women, Royal Women's Hospital, Victorian Cancer Registry; WA: King Edward Memorial Hospital, St John of God Hospitals Subiaco & Murdoch, Western Australian Cancer Registry.

SEARCH thanks the SEARCH research team for recruitment, and also acknowledges the assistance of the Eastern Cancer Registration and Information Centre for subject recruitment.

BECS thanks Reiner Strick, Silke Landrith and Sonja Oeser for their logistic support during the study.

CAHRES (formerly known as SASBAC) thanks Li Yuqing from the Genome Institute of Singapore for contributions to this study, and also acknowledges previous input to SASBAC resource creation by Anna Christensson, Boel Bissmarck, Kirsimari Aaltonen, Karl von Smitten, Nina Puolakka, Christer Halldén, Lim Siew Lan and Irene Chen, Lena U. Rosenberg, Mattias Hammarström, and Eija Flygare.

HJECS thanks Wen Zheng, Hermann Hertel, and Tjoung-Won Park-Simon at Hannover Medical School for their contribution to sample recruitment.

LES gratefully acknowledges Helena Soenen, Gilian Peuteman and Dominiek Smeets for their technical assistance.

MECS thanks Tom Sellers, Catherine Phelan, Andrew Berchuck, and Kimberly Kalli, Amanda von Bismarck, Luisa Freyer and Lisa Rogmann.

MoMaTEC thanks Britt Edvardsen, Ingjerd Bergo and Mari Kyllsø Halle for technical assistance and Inger Marie Aksnes and Tor Audun Hervig at the Blood bank, Haukeland University Hospital for assistance with control recruitment.

NECS thanks staff at the University of Newcastle and the Hunter Medical Research Institute.

NSECG thank Ella Barclay and Lynn Martin for their contribution, and acknowledge the invaluable help of the National Cancer Research Network with the collection of study participants.

RENDOCAS thanks Berith Wejderot, Sigrid Sahlen, Tao Liu, Margareta Ström, Maria Karlsson, and Birgitta Byström for their contribution to the study.

BSUCH thanks the Medical Faculty, Mannheim, the Diemtmar Hopp Foundation and the German Cancer Research Center.

MCCS was made possible by the contribution of many people, including the original investigators and the diligent team who recruited the participants and who continue working on follow up. We would like to express our gratitude to the many thousands of Melbourne residents who continue to participate in the study.

The UKBGS thank Breakthrough Breast Cancer and the Institute of Cancer Research for support and funding, and the Study participants, Study staff, and the doctors, nurses and other health care staff and data providers who have contributed to the Study. The ICR acknowledges NHS funding to the NIHR Biomedical Research Centre.

In addition, the iCOGS study would not have been possible without the contributions of: Andrew Berchuck (OCAC), Rosalind A. Eeles, Ali Amin Al Olama, Zsofia Kote-Jarai , Sara Benlloch (PRACTICAL), Georgia Chenevix-Trench, Antonis Antoniou, Lesley McGuffog, Fergus Couch and Ken Offit (CIMBA), Andrew Lee, and Ed Dicks, Craig Luccarini and the staff of the Centre for Cancer Genetic Epidemiology Laboratory (Cambridge), Javier Benitez, Anna Gonzalez-Neira and the staff of the CNIO genotyping unit, Jacques Simard and Daniel C. Tessier, Francois Bacot, Daniel Vincent, Sylvie LaBoissière and Frederic Robidoux and the staff of the McGill University and Génome Québec Innovation Centre, Stig E. Bojesen, Sune F. Nielsen, Borge G. Nordestgaard, and the staff of the Copenhagen DNA laboratory, Sharon A. Windebank, Christopher A. Hilker, Jeffrey Meyer and the staff of Mayo Clinic Genotyping Core Facility.

## E. Additional Acknowledgments of Funding to BCAC/OCAC control groups

The ESTHER study was funded by the Baden-Württemberg state Ministry of Science, Research and Arts (Stuttgart, Germany), the Federal Ministry of Education and Research (Berlin, Germany) and the Federal Ministry of Family Affairs, Senior Citizens, Women and Youth (Berlin, Germany).

The GENICA was funded by the Federal Ministry of Education and Research (BMBF) Germany grants 01KW9975/5, 01KW9976/8, 01KW9977/0 and 01KW0114, the Robert Bosch Foundation, Stuttgart, Deutsches Krebsforschungszentrum (DKFZ), Heidelberg, Institute for Prevention and Occupational Medicine of the German Social Accident Insurance, Institute of the Ruhr University Bochum (IPA), Germany, as well as the Department of Internal Medicine, Evangelische Kliniken Bonn gGmbH, Johanniter Krankenhaus, Bonn, Germany.

Financial support for the KARBAC study was provided through the regional agreement on medical training and clinical research (ALF) between Stockholm County Council and Karolinska Institutet, as well as the Swedish Cancer Society.

The MARIE study was supported by the Deutsche Krebshilfe e.V. [70-2892-BR I], the Hamburg Cancer Society and the German Cancer Research Center

MAY was supported by R01-CA122443, P50-CA136393, the Fred C. and Katherine B. Andersen Foundation, and the Mayo Foundation.

MCBCS recognizes funding from the Breast Cancer Research Foundation (BCRF), the David F. and Margaret T. Grohne Family Foundation, and the Ting Tsung and Wei Fong Chao Foundation

MCCS recruitment was funded by VicHealth and Cancer Council Victoria, and its follow-up has been continuously supported by infrastructure provided by Cancer Council Victoria.

UKBGS was funded by Breakthrough Breast Cancer and the Institute of Cancer Research, which acknowledges NHS funding to the NIHR Biomedical Research Centre
